# A comprehensive systematic review of sarcoptic mange diagnostic methods in wildlife

**DOI:** 10.7717/peerj.21609

**Published:** 2026-07-29

**Authors:** Chandni Sengupta, Julie M. Old

**Affiliations:** School of Science, University of Western Sydney, Hawkesbury, Penrith, New South Wales, Australia

**Keywords:** *Sarcoptes scabiei*, Free-ranging, Diagnosis, Skin scraping, Visual observation, Histological analysis, Mange scoring, PCR, Spotlighting, ELISA

## Abstract

Sarcoptic mange is a parasitic contagious disease that can lead to significant declines in wildlife populations. To identify the most used methods for diagnosing mange in free-ranging wildlife species, a systematic review was conducted across multiple databases: Scopus, PubMed, Web of Science, ProQuest Central and CABI. Citation screening was performed using the web-based platform, Covidence. A customized data extraction form, developed by the authors, was used to collect relevant information. This information was later exported and analyzed using Microsoft Excel. The objectives of this systematic review were to identify the most used detection methods for sarcoptic mange in wildlife globally over the past 30 years, classify them into invasive and non-invasive categories and provide an updated overview of related adjunct techniques and the broader scientific literature on mange detection and documentation in free-ranging animals. A total of 228 studies were analyzed and categorized into two groups—non-invasive and invasive methods. Non-invasive methods (visual observation, camera trapping, spotlighting, detector dogs and infrared thermography) do not require restraining of animals. Invasive methods (skin scraping, molecular diagnosis using mite isolation and polymerase chain reaction (PCR), serological diagnosis using Enzyme Linked ImmunoSorbent Assay (ELISA), histological analysis and immunohistochemistry) typically require taking samples from the animals and clinical or laboratory infrastructure. Across all diagnostic methods, visual observation was the most widely used technique, followed by skin scraping as the second most frequently applied method for confirming sarcoptic mange. Non-invasive approaches like spotlighting and camera traps provided valuable population-level insights without direct animal contact, though laboratory methods such as PCR, ELISA, and histological analysis offered greater accuracy at higher costs. We recommend a combined approach, starting with non-invasive methods to assess overall population health before using invasive techniques on selected animals for definitive diagnosis, with advanced technologies expected to enhance long-range detection capabilities in the future.

## Introduction

Sarcoptic mange (or scabies as it is known in humans) is caused by *Sarcoptes scabiei*, a parasitic mite belonging to the super order Acariformes and suborder Sarcoptiformes (Linnaeus, 1758) (Friedman, 1949; Pence & Ueckermann, 2002). The mite is consistently increasing its geographical distribution and number of host species and currently has the widest host range of any terrestrial parasite (Escobar et al., 2021; Pence & Ueckermann, 2002). At present, infestation by *S. scabiei* has been reported in at least 12 orders, 39 families and 148 mammalian species including both domestic and wild animals (Escobar et al., 2021). An outbreak of sarcoptic mange in naïve wildlife populations can result in a massive reduction in the number of individuals, as seen in Iberian ibexes (*Capra pyrenaica*) of Spain (León-Vizcaíno et al., 1999) and free-ranging red foxes (*Vulpes vulpes*) in Scandinavia (Mörner, 1992). Other major outbreaks have been reported in kit foxes (*Vulpes macrotis*) in California (Brian et al., 2017), black bears (*Ursus americanus*) in North America (Niedringhaus et al., 2019b), bare-nosed wombats (*Vombatus ursinus*) and koalas (*Phascolarctos cinereus*) in Australia (Skerratt, Martin & Handasyde, 1998; Speight et al., 2017), chamois (*Rupicapra rupicapra*) in northeastern Italy (Rossi et al., 1995) and raccoons (*Procyon lotor)* in Asia (Eo et al., 2008; Ninomiya & Ogata, 2005).

Severe outbreaks of this disease are often seen within small, genetically compromised or fragmented populations (Benson et al., 2019; Niedringhaus et al., 2019a; Pence et al., 1983). The increased vulnerability of populations is primarily attributed to reduced immunocompetence, fewer opportunities to mix with other populations that limit gene flow and recolonization potential, and the heightened impact of demographic stochasticity, which can further destabilize already vulnerable populations (Niedringhaus et al., 2019a; Pence & Ueckermann, 2002; Rousseau et al., 2021). *S. scabiei* infestations, therefore, have implications for mammalian wildlife species worldwide, particularly those that are endangered or threatened.

Sarcoptic mange is transmitted through direct or indirect transmission between animals. In animals, direct contact can occur during mating, fighting or allogrooming, whereas indirect transmission can be a result of the animal inhabiting dens or burrows of other infected animals (Escobar et al., 2021; Pérez et al., 1997; Rossi et al., 2007). Disease results from vigorous burrowing of the adult mites, laying of eggs and presence of mite allergens (such as mite feces and saliva), leading to intense skin irritation and formation of papular, vesicular, erythematous and pruritic lesions in the host animal (Arlian, 1989; Arlian & Morgan, 2017). Erythema and formation of parakeratotic scale further deteriorate leading to alopecia, excoriation and fissuring of parakeratotic scales (Skerratt, 2003). Significant energy demands and altered behavioural patterns are often associated with severe mange infestations (Cross et al., 2016; Simpson, Johnson & Carver, 2016). In general, hosts which are severely infected with *S. scabiei* die due to emaciation and secondary bacterial infections (Skerratt & Middleton, 1999).

Understanding the prevalence and impact of sarcoptic mange in wildlife requires accurate and efficient detection methods. Furthermore, before commencing treatment plans, it is of utmost importance to make the correct diagnosis. At present, many diagnostic approaches are used, some of which involve observing affected animals from a distance (such as camera trapping, visual observation through binoculars *etc.*), while others mandate restraining the affected individual and obtaining samples (such as skin scrapings, PCR (Polymerase Chain Reaction), histological analysis and ELISA (Enzyme-Linked Immunosorbent Assay)) (Angelone-Alasaad et al., 2015; Walton & Currie, 2007). In endemic settings, a preliminary diagnosis based on characteristic clinical signs is reasonable, particularly where mange is common, and other causes of alopecic or crusted lesions are less likely (Angelone-Alasaad et al., 2015; Niedringhaus et al., 2019a). However, definitive diagnosis of mange in wildlife relies on methods that directly detect the mite or its DNA such as microscopic examination of skin scrapings or molecular assays. Diagnosis of sarcoptic mange in wildlife is a challenge because most of these confirmatory techniques (*e.g* PCR, histology, ELISA) depend on specialized laboratory infrastructure, which may be inaccessible in remote areas (Walton & Currie, 2007). There is limited research into accurate and field-friendly diagnostic alternatives that balance feasibility, animal welfare and diagnostic certainty in these contexts. Lesions similar to those of sarcoptic mange are caused by other skin diseases as well as by other mange mites (Hardy, Engelman & Steer, 2017; Peltier et al., 2018) which makes diagnosis through visual observation difficult and sometimes misleading. In contrast, free-ranging animal sampling is complicated by factors such as elusive behaviour, protected status, nocturnal habits (Rojas et al., 2024), as well as logistical and regulatory constraints. Detection of sarcoptic mange may also be hampered by the limited persistence of mites on the host after death, which can be influenced by environmental conditions such as temperature and decomposition processes. However, few studies specifically address strategies to overcome these field-based constraints.

Additionally, species-specific differences in sarcoptic mange severity and host responses in free-ranging animals (Tiffin et al., 2025) complicate diagnosis. Current literature lacks comprehensive comparative studies or tailored protocols accounting for these differences between species.

Therefore, this systematic review aims to identify the most commonly used diagnostic methods for sarcoptic mange in wildlife, classify them into invasive and non-invasive categories and provide an updated overview of related adjunct techniques and the broader scientific literature on mange detection and documentation in free-ranging animals.

## Methodology

### Search strategy

A pilot search was carried out in Google Scholar to refine the search strategy before performing the full literature search. A systematic search of electronic databases PubMed, Embase *via* Ovid, Web of Science, Scopus, ProQuest central, Google Scholar, CABI, Mednar and BASE was conducted from 24/10/2023–14/11/2024 (see Table S1 for search queries applied). The search strategy was developed and conducted by the author (CS). Additionally, citations that were obtained through citation searching using the citationchaser application (Haddaway, Grainger & Gray, 2021), as well as manually checking the reference lists for additional publications, were included.

Search terms: “wild, undomesticated, free-ranging, free-roaming”, were included, while “domesticated, livestock, farm and captive animals” were excluded. A few studies mentioned both free-living and domestic animals; only data involving free-living species were extracted from these studies. Additionally, the search words included sarcoptic mange, assessment method, detection method, and diagnostic method. Truncation marks were combined to include all synonyms of the search terms. Authors restricted the search to exclude certain publication types, such as books or book chapters and review articles. Included studies were only in English. Only publications dated between 1992–2024 available in PubMed, Embase *via* Ovid, Web of Science, Scopus, ProQuest central, Google scholar, CABI, Mednar and BASE databases were included in this study. This date range has been chosen to focus on current methodologies and the recent development of diagnostic methods for identifying sarcoptic mange. Free-ranging mammals except humans were included in this study. Search terms used in all databases are provided in supplementary pages (Data S1).

### Citation screening and data extraction

Citation screening was conducted using the web-based software named Covidence (http://www.covidence.org/) by two reviewers (CS and JO). All citations obtained were transferred to EndNote 2020 which were then imported into Covidence as an XML file. Some duplicates were automatically removed by the software, while others required manual removal. Articles were screened by title and abstract. For articles eligible after initial screening, full texts were retrieved and reviewed by both authors. Studies deemed ineligible included those which were review articles, had the wrong study population (*e.g.*, captive or non-mammalian animals), were guidelines/recommendations, had full text inaccessible, had wrong disease type (*e.g.*, psoroptic mange), were not diagnostic, were not in English and were not published within the last thirty years. Discrepancies were resolved by consensus between authors (CS and JO). A Preferred Reporting Items for Systematic Reviews and Meta Analysis (PRISMA) flow diagram was generated using Covidence to document the screening process.

For data extraction, a form was created in Covidence by the authors to extract the relevant information. The categories included study identification, title of study, year of publication, country of study, publisher details, study area, year of study, study design, types of diagnostic methods used and population description. All relevant information was exported out of Covidence in .csv file and data analysis was carried out in Microsoft Excel for Microsoft 365 (Microsoft Corporation, Redmond, WA, USA).

### Data analysis

The dataset downloaded into Microsoft Excel was analyzed to determine the countries contributing most to sarcoptic mange in wildlife. The analysis also determined the most frequently studied free-ranging species and categorized them into their respective taxonomic orders and families. Additionally, the analysis revealed the types of environments where the studies were conducted, and the nature of the study design employed.

Types of diagnostic methods were identified and categorized into invasive and non-invasive groups. Invasive methods involved techniques which require animal handling in any form and non-invasive methods involved methods which do not require restraining and handling of animals. This classification of methods led the authors to identify the most popular methods used over time. A few adjunct methods were also identified which did not directly diagnose sarcoptic mange but helped in describing different attributes of the disease.

### Assessment of study quality and study classification

Following the data extraction process, quality of all articles was assessed using Scimago journal and country rank (https://www.scimagojr.com/) and only articles with a Q1 and Q2 ranking were included to ensure methodological rigor, as papers published in Q1 and Q2 journal typically demonstrate higher editorial and scientific standards compared to lower quartile journals. While journal quartile ranking does not assess the quality of individual study design, it provides a transparent proxy for baseline methodological standards of the publication venue. Grey literature was included only when it consisted of government reports or peer-reviewed academic theses. This approach ensured that only robust, well-validated data were incorporated while avoiding the exclusion of important wildlife-specific evidence not available in standard journal publications. Size of the study animal group was used to classify studies which had large (>50 animals), medium (20–50 animals), small (10–19 animals) or very small (<10 animals) as per Olivry & Mueller (2003).

## Results

**Figure 1:**
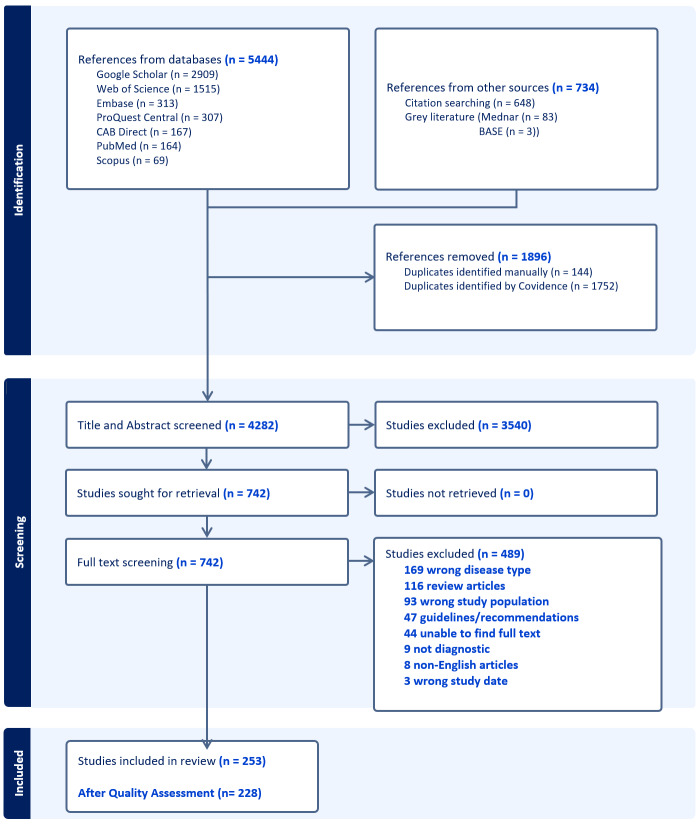
Flow diagram showing the steps during the screening process.

References (*n* = 5,444) were obtained from seven databases and 743 additional references were obtained from citation searching and grey literature. The title and abstract screening led to 742 studies being deemed eligible. Of these 742, 489 studies did not meet the full-text screening criteria and were removed. 253 studies met all eligibility standards and were extracted for data analysis. This multi-stage screening process ensured only relevant and eligible studies were included for data analysis (Fig. 1).

When classified based on size, most studies had a large sample animal population (*n* = 117), followed by very small (*n* = 36), medium (*n* = 29) and small (*n* = 11). Some articles included multiple species and were therefore categorized under multi-species studies, while a few others were categorized as “number of species not available” because the total number of species was not provided by the authors. Overall, 228 studies were included in the analysis after quality assessment.

Studies were carried out in 39 countries spanning all continents. Spain (*n* = 56) had the highest number of publications, followed by the USA (*n* = 39) and Australia (*n* = 35). Refer to Table S2 for additional details.

Animals belonging to Marsupialia (Diprodontia, Peramelemorphia, Dasyuromorphia, Didelphimorphia) and Eutheria (Artiodactyla, Carnivora, Lagomorpha, Primates (excluding humans), Eulipotyphyla, Rodentia) were investigated for the presence of sarcoptic mange. Most animals belonged to the order Carnivora, followed by Artiodactyla and Diprotodontia. Of all the animals investigated, one was critically endangered (red ruffed lemurs (*Varecia rubra*)), five were endangered (eastern quoll (*Dasyurus viverrinus*), reticulated giraffe (*Giraffa camelopardalis reticulata*), huemul deer (*Hippocamelus bisulcus*), European wild rabbit (*Oryctolagus cuniculus*), mountain gorilla (*Gorilla beringei beringei*)), while eight animals were vulnerable (koalas, Arabian oryx (*Oryx leucoryx*), goitered gazelle (*Gazella subguttarosa*), Chinese serow (*Capricornis sumatraensis*), barbary sheep (*Ammotragus lervia*), Nubian ibexes *(Capra nubiana*), eastern barred bandicoot (*Perameles gunnii*), cheetah (A*cinonyx jubatus)* and nine were near threatened (woylies (*Bettongia penicillata ogilvyi*), maned wolf (*Chrysocyon brachyurus*), Sechuran fox (*Lycalopes sechurae*), African buffalo (*Syncerus caffer*), Eurasian otter (*Lutra lutra*), European hedgehogs (*Erinaceus europaeus),* pudu (*Pudu puda)*, spotted-tailed Quoll (*Dasyurus maculatus),* European bison (*Bison bonasus)*) based on their IUCN (2025) status. Details available in Table S3.

In this review, remote locations refer to areas with minimal human presence or infrastructure typically characterized by restricted access and prioritized wildlife conservation—such as national parks, wildlife sanctuaries and protected forest reserves. With the exception of some studies conducted in urban (*e.g.*, Bakersfield campus and Taft, California state university) and semiurban areas (*e.g.*, Fire Island, USA), most studies took place in remote areas such as Sierra Nevada National Park in southern Spain, Bwindi Impenetrable National Park in Southwestern Uganda, Masoala National Park in northeastern Madagascar.

The common study designs included prevalence studies (*n* = 100), case reports (*n* = 69) and diagnostic accuracy studies (*n* = 16). All study design types are enlisted in Table S5.

Overall, data extraction identified two broad categories of mange detection methods: non-invasive and invasive methods. Most studies used a combination of non-invasive and invasive methods to confirm mange (Table S4). In non-invasive methods, the animals were observed and diagnosed from a distance without any intervention. These methods were of the following five types: visual observation, spotlighting, camera trapping, infrared thermography and detector dogs. Visual observation using handheld devices, such as binoculars or spotting scopes, allowed direct assessment of animals from a distance. Spotlighting consisted of nocturnal visual surveys using artificial light sources to observe free-ranging wildlife. Camera trapping involved the use of motion-activated cameras to remotely detect animals and identify clinical signs compatible with mange. Infrared thermography was applied to detect alterations in skin surface temperature potentially associated with inflammatory lesions. Detector dogs were trained to locate animals or carcasses showing signs of disease through olfactory cues.

Invasive methods required physically restraining the animals. These consisted of four types of methods: skin scraping, molecular diagnosis using mite isolation and PCR (polymerase chain reaction), serological diagnosis using ELISA (Enzyme Linked ImmunoSorbent Assay), histological analysis and immunohistochemistry Skin scraping technique involved collecting skin crusts through deep skin scraping to microscopically identify *S. scabiei* mites and eggs. Molecular diagnosis through mite isolation and PCR involved physically extracting mites from skin samples, often using flotation techniques or digestion methods to allow morphological identification and subsequent mite DNA analyses. Serological diagnosis through ELISA involved a blood-based method that measures host antibodies produced in response to *S. scabiei* infestation. Histological analysis involved identifying characteristic pathological changes associated with sarcoptic mange through microscopic examination of tissue sections. Immunohistochemistry is an extension of histology in which antibodies are tagged with markers that bind to mite antigens in tissue sections—this improves visualization and confirmation of *S. scabiei* when combined with histological assessment.

Our analysis identified additional techniques (mange scoring, historical data analysis, survey, radiotelemetry) which did not directly involve mange identification but served as methods to study important attributes of the affected population. Mange scoring involved a structured visual assessment system that grades the extent and severity of mange lesions on an animal, usually using standardized categorical scores. Historical data analysis involved the use of existing records (*e.g.*, past surveys, postmortem reports) to examine patterns and trends in sarcoptic mange occurrence over time and space. Radio telemetry involved a wildlife tracking technique that uses radio transmitters attached to animals to monitor their movements and behavior for sarcoptic mange related studies. Finally, survey-based approaches included citizen science reports, structured interviews with forest wardens and hunters, and data collected through public reporting platforms.

**Figure 2:**
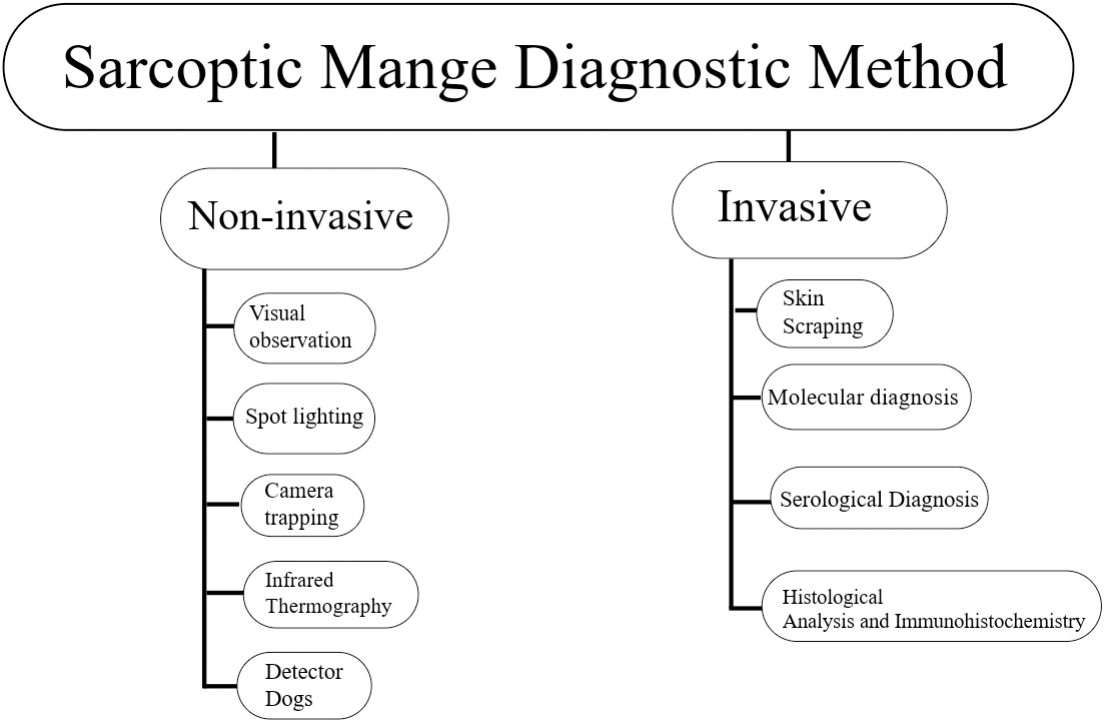
Categories and subcategories of mange diagnostic techniques.

The most popular method of sarcoptic mange identification occurred through visual observation with 141 studies using this detection method, followed by skin scraping (*n* = 133), histopathology and immunohistochemistry analysis (*n* = 46), mite isolation and PCR (*n* = 42), ELISA (*n* = 29), camera traps (*n* = 27), spotlighting (*n* = 9), infrared thermography (*n* = 1) and detector dogs (*n* = 1) (Fig. 2).

## Discussion

Overall, we analyzed 228 studies to determine the most common method used in the field to identify sarcoptic mange in wildlife based on our eligibility criteria. The studies spanned all continents and included ninety different species of animals. All animals included in this study were free-ranging; however, the studies included animal populations from not only remote areas but also those occurring in urban and semi-urban locations.

### Non-invasive methods

Factors such as co-infestations with different pathogens, overall health, and the genetic makeup of both the host and the mites are additionally responsible for variation in infestation severity and duration (Brandell, 2021). Furthermore, during early infestation, when the signs such as hair loss or crust formation are absent or subtle, a diagnosis using this method may be misleading. Accordingly, these methods record low sensitivity and specificity during the initial mange infestation stages (Fraser et al., 2018b). Additionally, diagnosis through visual assessment can be potentially unreliable when investigating animals with dense fur coats, which can obscure lesions. Therefore, a customized sarcoptic mange identification protocol for each species is needed that takes into consideration species-specific anatomy, behaviour, and disease patterns. Underestimating the actual disease prevalence and possibly producing false epidemiological conclusions are two consequences of this detection bias. Consequently, without supplementary diagnostic techniques, non-invasive diagnostic methods cannot offer conclusive management confirmation. Wails et al. (2024) specifically caution against relying solely on camera trap data for confirming sarcoptic mange outbreaks in red foxes, emphasizing the necessity of integrating confirmatory methods such as skin scraping or PCR analysis for accurate disease verification. This requirement for supplementary testing reduces the method’s standalone utility and increases overall surveillance costs and complexity.

Nevertheless, these methods allow detection and monitoring of affected populations without capturing or handling animals, which reduces stress and potential harm to wildlife (Grandin, 1997). These protocols not only are cost effective but also enable broad population level surveillance (visual observation, spotlighting and camera traps). Spotlighting and infrared thermography allow monitoring of elusive and nocturnal species.

#### Visual observation

Visual observation was the most popular sarcoptic mange diagnostic technique. Most researchers preferred this method because it can be implemented across a wide range of species (Table S6B) in remote locations, where application of other methods may be challenging. For example, Pérez et al. (2011) employed park rangers to visually assess Iberian ibex using binoculars at Sierra Nevada National Park, categorizing animals based on the extent of affected skin area. Suspected cases were later culled and confirmed through skin scraping analysis.

Building on basic visual diagnosis, several studies applied a scoring system to quantify the severity of sarcoptic mange in affected populations known as mange scoring (Table S6C). These scoring systems, such as those developed by Borchard, Eldridge & Wright (2012) and later modified by Simpson, Johnson & Carver (2016), provide consistent evaluation criteria based on visual symptoms, including erythema (skin reddening), parakeratosis (skin thickening), and alopecia (hair loss). Similar severity scores have been used in studies of bare-nosed wombats in New South Wales, Australia (Sengupta, 2019; Stannard et al., 2021) allowing comparison of disease burden within and between populations over time. Sensitivity and specificity of visual diagnosis are likely to vary across species and environmental contexts. For example, in free-ranging Iberian ibex, visual diagnosis shows a the sensitivity of 87.14% and a moderate specificity of 60.71% with performance differing by age, sex and season (Valldeperes et al., 2019).

#### Spotlighting

Spotlighting (Table S6D) is typically used for mange detection in nocturnal animals such as red foxes (Gortázar et al., 1998) and bare-nosed wombats (Casey et al., 2024) where vehicle-based nocturnal surveys correspond with natural activity patterns. This approach is highly accessible and practical, since it does not require complex machinery or laboratory facilities, making it suitable for remote field locations. Investigations by Stannard et al. (2021) and Burgess et al. (2023) have successfully implemented spotlighting over several Australian sites, demonstrating its practical reliability when carried out by skilled professionals using standardized mange scoring charts. However, reliability of visual severity scoring depends on the observer’s experience and their familiarity with the scoring system (Skerratt et al., 2021; Stannard et al., 2021). Spotlighting has seen limited research adoption, with only nine studies identified using this technique in our review, which may reflect methodological constraints or lower accuracy compared to other detection methods.

#### Camera trap

Camera traps (Table S6F) not only offer a non-invasive way to monitor the health of wildlife, but are analyzed to get the apparent mange prevalence, which is defined as the proportion of camera trapping events of red foxes with mange-compatible lesions over the total number of red foxes (Sanchez et al., 2017). Furthermore, camera trapping can be used to document disease progression in individuals. However, in one study (Brewster et al., 2017), a veterinarian was able to correctly identify the mange status of 12 species in 69% of photographs, with 17% misidentified and 14% inconclusive, demonstrating moderate reliability for photographic diagnosis.

Long-term tracking of disease progression and population health dynamics is made possible by this methodology. A study carried out in Alt Pirineu National Park, Spain (Barroso & Palencia, 2024) visually assessed camera trap photographs of red foxes and determined that the apparent prevalence in this fox population was severe.

A key limitation of camera trapping is the difficulty of reliably distinguishing the individual animals from images, particularly where natural markings are subtle or photographs are blurred, which can lead to misidentification and biased abundance estimates (Dorning & Harris, 2019; Johansson et al., 2020). To overcome the limitations of population estimation using camera traps, and the limitations of population estimation, Barroso & Palencia (2024) adopted a standardized approach based on independent detection events (>10 min apart) and used Relative Abundance Index instead of true population size. Data reliability was improved by excluding camera traps with low detection numbers, while potential interactions among individuals were inferred through spatial coincidence with a 24 h window.

#### Infrared thermography

Infrared thermography as a diagnostic tool was investigated by Arenas et al. (2002). The authors evaluated the effectiveness of infrared thermal imaging for diagnosing sarcoptic mange in free-ranging Iberian ibex, comparing two diagnostic approaches: conventional binocular observation and infrared thermography. The study found that conventional binocular observation demonstrated superior sensitivity and specificity compared to infrared thermography for remote diagnosis of sarcoptic mange for this species (initial and epidemic phases). Although infrared thermography enables non-invasive, remote detection of infestation, its sensitivity and specificity are significantly reduced at distances exceeding 100 m (Arenas et al., 2002). Therefore, the authors recommended not considering infrared thermography as the most reliable tele-diagnostic tool for sarcoptic mange.

#### Detector dogs

A rare method of mange detection in the wild is the use of detector dogs. Alasaad et al. (2012), successfully trained two Bavarian Mountain hound dogs to locate carcasses of mangy animals and identify and capture live animals suspected of sarcoptic mange infestation. These detector dogs were able to localize 292 mangy carcasses and 63 live mange-infected animals in the Italian Alps over 15 years. All carcasses and live animals were confirmed to have sarcoptic mange on postmortem examination and no false positives were reported. However, the overall sensitivity and specificity of this method could not be estimated because the total number of infected individuals in the population was unknown and the authors emphasized the need for further controlled studies to quantify diagnostic accuracy. To date, detector dogs have been reported for sarcoptic mange in only one study, suggesting potential constraints such as the need for specialized training, ongoing maintenance of detector dogs and suitable field conditions. Nevertheless, this technique is relatively non-invasive, assuming it does not disturb wildlife and alter animal behaviour, and appears effective for detecting mange in both dead and live animals across large, remote areas.

#### Historical data analysis

Several studies utilized historical data as an additional technique to understand the characteristics of the sarcoptic mange affected populations. Analysis of past literature helped in identifying important vectors of mange, for example, raccoon dogs serve as critical vectors of mange in Lithuania (Janulaitis et al., 2014). Akdesir et al. (2018) analyzed necropsy reports of mustelids at the University of Bern, Switzerland, from 1958 to 2015, providing exceptional temporal coverage but limited to mortality data. A similar study by Rojas-Sereno, Abbott & Hynes (2022) used necropsy data from 2009 to 2018 on dead or euthanized American black bears to compare the data with visual observation data and assess the contemporary status of mange in the bear population. Table S6L lists all studies that used historical analysis as a part of their analysis. Historical data analysis can be a source of information about a diverse range of species, provided the databases are well maintained, which is subject to consistent data collection procedures throughout the study period. The benefits of this method are its cost-effectiveness and its ability to analyze long-term epidemiological patterns. However, researchers should be aware of the potential data gaps and inconsistent historical recording practices.

#### Survey

Survey-based approaches (Table S6E) and citizen science observations are tools for documenting the occurrence and spatio-temporal trends of sarcoptic mange, particularly in remote regions with dense vegetation, where closely examining animals is logistically challenging. Information provided by park rangers, citizen scientists and indigenous communities can represent the only feasible means of documenting mange and thus can offer original insights into its presence and spread. Chen et al. (2012) interviewed tribal communities and park rangers in the Central and Coastal mountain range of Taiwan to understand the distribution and prevalence of sarcoptic mange in wild Formosan serow (*Capricornis swinhoei*). Large-scale data collection is exemplified by the Wombat Survey and Analysis Tool (WomSAT), which captured 22,000 bare-nosed wombat sightings with mange severity scores, enabling landscape-level analysis of sarcoptic mange distribution patterns (Mayadunnage, Stannard & West, 2023). The approach successfully integrates multiple data sources, from forestry departments monitoring alpine chamois long-term demographic trends (Obber et al., 2022) to utilizing data reported by citizens in digital news, social media and other internet-based platforms for estimating mange prevalence in Chilean wild mammals (Montecino-Latorre et al., 2020) as well as using platforms like iNaturalist and Ornitho for red fox mange assessment (Franchini et al., 2022). Additionally, these surveys offer important ecological insights by identifying trends like increased occurrence in urban areas and a higher prevalence of mange in juvenile foxes compared to adult foxes (Franchini et al., 2022). However, this technique is largely dependent on visual evaluation by non-experts, which introduces significant subjectivity and the possibility of misclassification (Resnik, Elliott & Miller, 2015). Data sources can differ in terms of reliability, ranging from social media observations by the general public to trained park rangers. However, by using iterative project design, appropriate and standardized volunteer protocols, thorough data analysis techniques, metadata capture, and exacting accuracy assessment, researchers can improve the quality of their data (Kosmala et al., 2016).

### Invasive methods

Direct techniques like skin scraping and PCR provide reliable confirmation and allow detection of mild and atypical or subclinical cases. PCR and ELISA can detect past infestations; however, these methods may also produce rare false positives, as mite DNA and antibodies can persist in the host even after the infestation has resolved (Ráez-Bravo et al., 2016; Yang, Kim & Kim, 2021). Laboratory-based methods can distinguish between mite species, enhancing diagnostic specificity and accuracy of *Sarcoptic scabieii* detection. Some invasive protocols (skin scraping and scoring mite burden, histological analysis, PCR) allow quantification of mites and detection of co-infections/multiple pathogens. Studies on humans suggested using ELISA along with PCR to make detection more sensitive, especially in patients with low mite burden (Bezold et al., 2001).

#### Skin scraping

Our analysis identified skin scraping (alone) to be the most popular invasive method (*n* = 44), likely because it enables direct identification of *S. scabiei* and often facilitates relevant treatment (Arzamendia et al., 2012; Horowitz et al., 2020). Furthermore, we confirmed that skin scraping has been successfully applied across diverse animal species, demonstrating its broad applicability and non-species-specific nature (Table S6A). However, the sensitivity of skin scraping can be low (50%) when few mites are present or when a skilled practitioner does not perform the procedure (Walton & Currie, 2007).

In general, deep skin scrapings were taken from suspected cases (Foley et al., 2023) or carcasses (Domínguez et al., 2008) using a sterile surgical scalpel, which were then examined under a dissecting microscope for the presence of *S.scabiei* mite (Millán et al., 2024)—unless some studies digested the skin scrapings with potassium hydroxide (Klink et al., 2023) or mounted the skin scrapings on a slide with liquid paraffin (Skorupski et al., 2022). Mites were identified using a diagnostic reference (morphologic features of *S. scabiei* as found in Fain (1968). Additionally, several studies employed a scoring system to categorize mite burden as demonstrated in a study of American black bears (Tiffin et al., 2024) with scoring ranges from 0–3 based on mite counts.

#### Molecular diagnosis—mite isolation and PCR

Molecular diagnosis through PCR is usually done on skin scrapings or on swabs taken from the affected area of the infected individual. Fraser et al. (2018b) found that PCR on skin scrapings had higher sensitivity (100%) and specificity (84.62%) than PCR on swabs. For PCR on skin samples *S. scabiei* mites are often first retrieved from scabietic skin through any of the following methods: mite isolation (Bornstein, 1995; Sheahan & Hatch, 1975), post-frozen mite isolation (Alasaad et al., 2009), syringe Eppendorf tube method (Soglia et al., 2009), sedimentation and direct floatation (Bojarska et al., 2024) and a microtube method (Kido et al., 2017) (Table S6K). Of these, researchers have used the post-frozen mite isolation method more frequently than the other mite isolation methods. Mite isolation techniques do not constitute diagnostic tests on their own but serve as preparatory procedures to obtain sarcoptic mange mites or mite material for preparation of purified antigen for serological assays or experimental infestations in controlled settings or PCR-based assays. For PCR based assays, isolated mites are observed under a microscope for morphological confirmation and used as an input to confirm *S.scabiei* infestation.

PCR has frequently been used as a diagnostic tool for mange detection (Montecino-Latorre et al., 2020), while downstream analyses (*e.g.*, sequencing and microsatellite genotyping of PCR amplicons) have been applied to understand origin of infestations and population structure of *S. scabiei*. Viani et al. (2023) and Moroni et al. (2023) did not rely on simple PCR alone, but used microsatellite markers to characterize the genetic profiles of sarcoptic mange mites. Viani et al. (2023) analyzed 33 *S. scabiei* mites from seven wild boars and eight red foxes in the Aosta Valley, Italy and explored whether mange emergence in wild boars was linked to prey-predator transmission or within-species transmission. Given the small sample size, the authors considered both predator–prey and within-species transmission plausible and did not draw definitive conclusions about transmission dynamics. Similarly, Moroni et al. (2023) examined mites from wild and domesticated felids in Italy and neighbouring countries and found closely related mite genotypes are shared among affected felids and sympatric canids. Their findings suggest that mite population structure was more strongly tied to shared geographic proximity than to host species.

Importantly, research on the mitochondrial genome sequencing of *S.scabiei* revealed that even within single hosts, mites exhibit unique haplotypes with extremely high similarity but not clonality, suggesting that the mites differ at the level of individual genetic variants rather than forming distinct strains (Fraser et al., 2017). Table S6G lists all studies identified by our analysis that employed PCR to detect mange and, in some cases, to conduct further genetic characterization of *S. scabiei*.

#### ELISA

ELISA performance for sarcoptic mange diagnosis varies substantially depending on the specificity and cross-reactivity of the antibodies used, particularly when assays validated for one species are applied to others. For example, Ráez-Bravo et al. (2016) evaluated three ELISAs for sarcoptic mange diagnosis in Iberian ibex, with mange confirmed by visual assessment and scoring. The ELISA originally developed for alpine chamois detected specific antibodies (IgG) to mange in Iberian ibex sera with higher sensitivity (93%) and specificity (93.5%) than assays developed for dogs or Cantabrian chamois (*R. pyrenaica parva*). This chamois-validated ELISA was used as a screening tool in later Iberian ibex studies, including work by Valldeperes et al. (2024), to distinguish mange affected Ibexes from apparently healthy animals prior to further ecological analyses. Table S6H lists all studies identified by our analysis.

ELISA techniques are particularly valuable for detecting asymptomatic carriers or animals recovered from past infestations, as circulating antibodies to mange indicate previous exposure to the disease (Peltier et al., 2018). However, cross-species application requires thorough validation, and seropositivity cannot distinguish active from resolved infestations.

#### Histological analysis and immunohistochemsitry

Histopathology of skin biopsies has been used as a diagnostic tool in a few studies (Knowles et al., 2017; Oleaga et al., 2018). In contrast, most studies used histological analysis in combination with other confirmatory tests (Table S6I) to confirm sarcoptic mange and to characterize associated lesions and host responses. Nimmervoll et al. (2013) applied a combination of methods to detect mange in free-ranging foxes in Switzerland. ELISA, mite isolation and histological analysis were used as confirmatory tests for sarcoptic mange. Skin sections of healthy and mangy foxes were categorized as zero to one based on the severity of the disease using histological features such as thickness of crust, alopecia, number of mites and eosinophil, lymphocyte and mast cell counts. Similarly, Thrivikraman et al. (2023) used histological analysis as a confirmatory test to diagnose mange in in Arabian oryx carcasses in Dubai in addition to skin scraping.

In addition to conventional staining, immunohistochemistry has been identified as a valuable adjunct, particularly in free-ranging animals with low burdens, where mites may be difficult to visualize in routine sections. Oleaga et al. (2012) suggested that immunohistochemistry can serve as a useful alternative or complement for diagnosing sarcoptic mange when other diagnostic methods are unavailable, by highlighting mite antigens and associated inflammatory cells within lesional skin. Immunohistochemical approaches have also been applied for more detailed lesion analysis in species inhabiting diverse habitats like wild boars (Valldeperes et al., 2021) and alpine chamois (Salvadori et al., 2016). Although our analysis found only three studies incorporating immunohistochemistry into their detection protocol (Table S6M), these findings indicate that it can function as a dependable extension of histology, especially in wildlife populations where standard diagnostic techniques are logistically challenging.

#### Radio telemetry

Radio telemetry was used as an adjunctive method to gain valuable behavioral and spatial context for sarcoptic mange rather than to diagnose infestation directly. Using collared San Joaquin kit foxes, Cypher et al. (2023) combined telemetry data with the disease status to demonstrate that den sharing could facilitate sarcoptic mange transmission through overlapping spatiotemporal use of dens. In a related study by Cypher & Deatherage (2023), the authors monitored movements of San Joaquin kit foxes and concluded that kit foxes with sarcoptic mange were less active than those without mange. These examples illustrate how telemetry can complement diagnostic methods by revealing contact structure, movement patterns, and potential transmission pathways in affected populations. Table S6J lists all studies that were identified by our analysis that used radio telemetry.

All invasive methods require capture, physical handling and may include euthanasia or tissue biopsy, thereby presenting significant concerns regarding animal welfare and ethical standards. For instance, histological analysis mandates invasive biopsy procedures, limiting its application in live animals. Haas et al. (2015) in their study on wild boars of Switzerland underscored that non-invasive tools such as camera traps, surveys and syndromic surveillance can be used to pinpoint suspected cases of mange from a whole population while invasive tests such as histological analysis should be reserved for a limited number of individuals to confirm mange in those suspected cases within the affected population.

Invasive diagnostic techniques depend heavily on operator skills. Skin scraping is heavily reliant on the skill and expertise of the individual performing this procedure. The success of detection by skin scraping is dependent on precise identification of optimal scraping locations, which may not be obvious in the early stage of infestation (Peltier et al., 2018). Consequently, multiple skin scrapings are recommended to improve mite detection using this method (Miller, Griffin & Campbell,, 2012).

Invasive diagnostic protocols are resource-intensive, time-consuming and expensive. PCR is highly sensitive and effective; however, its limitations include the requirement for specialized equipment and expertise, which may not be readily available in all settings—hindering its widespread use as a diagnostic tool (Bae et al., 2020; Fraser et al., 2018a). However, modern equipment allows PCRs to be conducted in the field. Fraser et al. (2018a), suggested a benchtop method, Loop Mediated Isothermal Amplification (LAMP) assay, which analyzed clinical samples from various hosts targeting the ITS-2 gene for *S. scabiei*. The authors cautioned that this method may result in cross-contamination and misleading results.

Invasive diagnostic techniques such as skin scraping have sensitivity limitations. The effectiveness of skin scraping diminishes when mite populations are low, particularly in mild or asymptomatic cases (Pence & Ueckermann, 2002). This detection technique can prove inadequate for animals with delayed hypersensitivity reactions, where severe itching and skin trauma occur despite reduced mite burdens (Pence & Ueckermann, 2002).

## Conclusion

Sarcoptic mange diagnosis in wildlife has been conducted through non-invasive and invasive methods, each with its own specific advantages and limitations. The authors recommend employing non-invasive methods such as visual observation, spotlighting, camera traps, and surveys for wildlife population surveillance while restricting invasive protocols such as skin scraping, PCR, ELISA, and histological analysis to a limited number of suspected individuals for confirmation of sarcoptic mange. Advanced technologies will likely improve detection of small lesions at long range.

##  Supplemental Information

10.7717/peerj.21609/supp-1 Supplemental Information 1 Supplemental Data showing the search queries from all databases, list of countries, list of animals, and different diagnosis methods used in studies

10.7717/peerj.21609/supp-2 Supplemental Information 2 Prisma 2020 Checklist

10.7717/peerj.21609/supp-3 Supplemental Information 3 Intended audience

## Additional Information and Declarations

### Data Availability

The following information was supplied regarding data availability: This is a Systematic review/Meta-analysis.
