# Supplementary material for: A comprehensive systematic review of sarcoptic mange diagnostic methods in wildlife
Source: PeerJ. 2026 Jul 29;14:e21609. doi: 10.7717/peerj.21609 (PMC13428544; doi:10.7717/peerj.21609)
Supplement: Supplemental Information 1 [file peerj-14-21609-s001.docx]

S1: Search query:

**Scopus**

TITLE-ABS ( ( assess* OR detect* OR diagnosis ) AND ( sarcoptic AND mange OR mange* OR scabiei ) AND ( wild OR field OR free-roaming OR free-ranging OR undomesticated ) ) AND PUBYEAR > 1992 AND PUBYEAR < 2024 AND ( LIMIT-TO ( DOCTYPE , "ar" ) OR LIMIT-TO ( DOCTYPE , "ch" ) ) AND ( LIMIT-TO ( LANGUAGE , "English" ) )

**Embase Ovid**

((asses* or detect* or diagnose*) and (sarcopt* or Scab* or mange*) and (wild* or Free-ranging or Free-roaming or undomesticated or field)).ti,ab. limit 1 to (english language and yr="1993 - 2024" and (article or article in press or books or chapter or conference abstract or conference paper or letter or note or "preprint (unpublished, non-peer reviewed)" or short survey))

**PubMed**

(((("assess"[All Fields] OR "assessed"[All Fields] OR "assessement"[All Fields] OR "assesses"[All Fields] OR "assessing"[All Fields] OR "assessment"[All Fields] OR "assessment s"[All Fields] OR "assessments"[All Fields] OR ("detect"[All Fields] OR "detectabilities"[All Fields] OR "detectability"[All Fields] OR "detectable"[All Fields] OR "detectables"[All Fields] OR "detectably"[All Fields] OR "detected"[All Fields] OR "detectible"[All Fields] OR "detecting"[All Fields] OR "detection"[All Fields] OR "detections"[All Fields] OR "detects"[All Fields]) OR ("diagnosable"[All Fields] OR "diagnosi"[All Fields] OR "diagnosis"[MeSH Terms] OR "diagnosis"[All Fields] OR "diagnose"[All Fields] OR "diagnosed"[All Fields] OR "diagnoses"[All Fields] OR "diagnosing"[All Fields] OR "diagnosis"[MeSH Subheading])) AND ((("sarcoptes"[All Fields] OR "sarcoptic"[All Fields]) AND ("mite infestations"[MeSH Terms] OR ("mite"[All Fields] AND "infestations"[All Fields]) OR "mite infestations"[All Fields] OR "mange"[All Fields])) OR ("mite infestations"[MeSH Terms] OR ("mite"[All Fields] AND "infestations"[All Fields]) OR "mite infestations"[All Fields] OR "mange"[All Fields]))) OR "scabiei"[All Fields]) AND ("wild"[All Fields] OR ("field"[All Fields] OR "field s"[All Fields] OR "fields"[All Fields]) OR "free-roaming"[All Fields] OR "free-ranging"[All Fields] OR "undomesticated"[All Fields])) AND ((booksdocs[Filter] OR casereports[Filter] OR clinicalconference[Filter] OR clinicaltrial[Filter] OR comparativestudy[Filter] OR correctedandrepublishedarticle[Filter] OR letter[Filter] OR meta-analysis[Filter] OR veterinaryobservationalstudy[Filter] OR preprint[Filter] OR randomizedcontrolledtrial[Filter] OR researchsupportnihextramural[Filter] OR researchsupportnihintramural[Filter] OR researchsupportnonusgovt[Filter] OR researchsupportusgovtnonphs[Filter] OR researchsupportusgovtphs[Filter] OR researchsupportusgovernment[Filter] OR technicalreport[Filter] OR validationstudy[Filter]) AND (animal[Filter]) AND (1992/12/31:2024/1/1[pdat]) AND (english[Filter]))

**Web of science**

((TS=("assess" OR "detect" OR "diagose") AND TS=("sarcop" OR "mange" OR "scabiei") AND TS=("wild" OR "field" OR "undomesticated" OR "free-ranging" OR "free-roaming")) OR (QMTS=("SARCOPTIC MANGE")) OR (QMTS=("SARCOPTES SCABIEI")) OR (QMTS=("MANGE")) OR (QMTS=("SARCOPTES"))) AND ((DT==("ARTICLE" OR "CASE REPORT" OR "LETTER" OR "DISSERTATION THESIS" OR "PREPRINT" OR "BOOK" OR "CLINICAL TRIAL") AND SILOID==("PPRN" OR "WOS" OR "MEDLINE" OR "CCC" OR "PQDT" OR "KJD" OR "SCIELO" OR "CSCD" OR "PPRN") AND LA==("ENGLISH")) NOT (DT==("REVIEW") OR ORG==("SARCOPTES SCABIEI VAR HOMINIS" OR "DEMODEX BREVIS" OR "DEMODEX FOLLICULORUM" OR "ECHINOCOCCUS MULTILOCULARIS" OR "DEMODEX" OR "IXODES SCAPULARIS" OR "IXODES TASMANI" OR "LEISHMANIA INFANTUM" OR "NEMATODIRUS" OR "PSOROPTES EQUI" OR "PSOROPTES CERVINUS" OR "PSORERGATIDAE" OR "PSOROPTIDAE" OR "TOXASCARIS LEONINA" OR "TOXOCARA") OR SJ==("GEOLOGY" OR "LINGUISTICS" OR "SOCIAL WORK" OR "ACOUSTICS" OR "BIOPHYSICS" OR "FILM RADIO TELEVISION" OR "HISTORY PHILOSOPHY OF SCIENCE" OR "LITERATURE" OR "PHYSICAL GEOGRAPHY" OR "RELIGION" OR "WOMEN S STUDIES" OR "CRIMINOLOGY PENOLOGY" OR "GEOCHEMISTRY GEOPHYSICS" OR "OPTICS" OR "SPORT SCIENCES" OR "ART" OR "CONSTRUCTION BUILDING TECHNOLOGY" OR "CULTURAL STUDIES" OR "MECHANICS" OR "PHILOSOPHY" OR "PHYSICAL SCIENCES OTHER TOPICS" OR "ROBOTICS" OR "SUBSTANCE ABUSE" OR "AREA STUDIES" OR "AUDIOLOGY SPEECH LANGUAGE PATHOLOGY" OR "FISHERIES" OR "OCEANOGRAPHY" OR "PALEONTOLOGY" OR "THEATER" OR "TRANSPORTATION" OR "WOMEN APOS S STUDIES" OR "ARCHAEOLOGY" OR "CRYSTALLOGRAPHY" OR "METALLURGY METALLURGICAL ENGINEERING" OR "POLYMER SCIENCE" OR "SPECTROSCOPY" OR "ETHNIC STUDIES" OR "TRANSPLANTATION" OR "INTERNATIONAL RELATIONS" OR "ARTS HUMANITIES OTHER TOPICS" OR "REHABILITATION" OR "MEDICAL ETHICS" OR "LEGAL MEDICINE" OR "ENERGY FUELS" OR "EMERGENCY MEDICINE" OR "AUTOMATION CONTROL SYSTEMS" OR "MATHEMATICAL COMPUTATIONAL BIOLOGY" OR "COMPUTER SCIENCE" OR "METEOROLOGY ATMOSPHERIC SCIENCES" OR "SOCIAL ISSUES" OR "ENGINEERING" OR "ONCOLOGY" OR "UROLOGY NEPHROLOGY" OR "NURSING" OR "INFORMATION SCIENCE LIBRARY SCIENCE" OR "HISTORY" OR "PSYCHIATRY" OR "COMMUNICATION" OR "URBAN STUDIES" OR "OBSTETRICS GYNECOLOGY" OR "RHEUMATOLOGY" OR "ORTHOPEDICS" OR "PUBLIC ADMINISTRATION" OR "RADIOLOGY NUCLEAR MEDICINE MEDICAL IMAGING" OR "SOCIAL SCIENCES OTHER TOPICS" OR "DENTISTRY ORAL SURGERY MEDICINE" OR "FAMILY STUDIES" OR "MYCOLOGY" OR "MATERIALS SCIENCE" OR "OPHTHALMOLOGY" OR "GEOGRAPHY" OR "PHYSICS" OR "WATER RESOURCES" OR "MARINE FRESHWATER BIOLOGY" OR "TELECOMMUNICATIONS" OR "SOCIOLOGY" OR "CARDIOVASCULAR SYSTEM CARDIOLOGY" OR "EDUCATION EDUCATIONAL RESEARCH" OR "NEUROSCIENCES NEUROLOGY" OR "BUSINESS ECONOMICS" OR "PSYCHOLOGY" OR "GASTROENTEROLOGY HEPATOLOGY" OR "NUTRITION DIETETICS" OR "REPRODUCTIVE BIOLOGY" OR "GENERAL INTERNAL MEDICINE" OR "MATHEMATICS" OR "GERIATRICS GERONTOLOGY" OR "PEDIATRICS" OR "PUBLIC ENVIRONMENTAL OCCUPATIONAL HEALTH")))

**CAB direct**

((asses* or detect* or diagnos*) AND ab:(sarcopt* or mange or scabie ) AND (wild* or free-roaming or free-ranging or undomesticated or field) AND yr:[1992 TO 2024]) AND ( ((NOT (language:(( "Portuguese" OR "Japanese" OR "Finnish" OR "Spanish" OR "German" OR "Danish" OR "Chinese" OR "Indonesian" OR "French" OR "Italian" OR "Dutch" OR "Polish" ) ))) (NOT (organism-descriptor:(( "man" ) ))) (NOT (topic:(( "domestic animals" OR "livestock" ) ))) ))

**Proquest**

((asses* OR detect* OR diagnos*) AND (sarcopt* OR mange OR scabi*) AND (wild* OR free-ranging OR free-roaming OR undomesticated OR field) AND stype.exact("Conference Papers & Proceedings" OR "Government & Official Publications" OR "Reports" OR "Books" OR "Working Papers" OR "Scholarly Journals" OR "Dissertations & Theses") AND at.exact("Book Chapter" OR "Research Topic" OR "Annual Report" OR "Dissertation/Thesis" OR "Government & Official Document" OR "Working Paper/Pre-Print" OR "Conference Paper" OR "Conference" OR "Technical Report" OR "Evidence Based Healthcare" OR "Transcript" OR "Report" OR "Statistics/Data Report" OR "Case Study" OR "Conference Proceeding" OR "Country Report") AND la.exact("English") AND pd(19921231-20240101)) NOT (subt.exact("public health" OR "acquisitions & mergers" OR "venture capital" OR "private equity" OR "capital formation" OR "equity stake" OR "securities offerings" OR "womens studies" OR "cultural anthropology" OR "sociology" OR "romance literature" OR "management" OR "clinical psychology" OR "european history" OR "nursing" OR "financial statements" OR "asset acquisitions" OR "linguistics" OR "political science" OR "literature" OR "history" OR "individual & family studies" OR "comparative literature" OR "computer science" OR "american history" OR "employees" OR "social psychology" OR "licensed products" OR "american literature" OR "educational administration" OR "agreements" OR "neurosciences" OR "higher education" OR "school administration" OR "alliances" OR "licenses" OR "communication" OR "british & irish literature" OR "modern literature" OR "philosophy" OR "psychology" OR "education" OR "medical device industry" OR "religion" OR "mental health" OR "psychotherapy" OR "developmental psychology" OR "families & family life" OR "ethnic studies" OR "educational leadership" OR "educational psychology" OR "public policy" OR "art history" OR "agronomy" OR "language" OR "social work" OR "british and irish literature" OR "chemistry" OR "science history" OR "geography" OR "curriculum development" OR "economics" OR "cognitive psychology" OR "teaching" OR "public administration" OR "social structure" OR "minority & ethnic groups") NOT la.exact("FRE" OR "TUR" OR "SPA" OR "LAT") AND PEER(yes)) AND pd(19921231-20240101)

**Google Scholar**

sarcoptic mange asses AND wild OR "free ranging" OR free-roaming or undomesticated OR field

limits: date range -2019-2024

Table S2: Table listing countries that employed mange diagnosis methods along with the publication year of each study


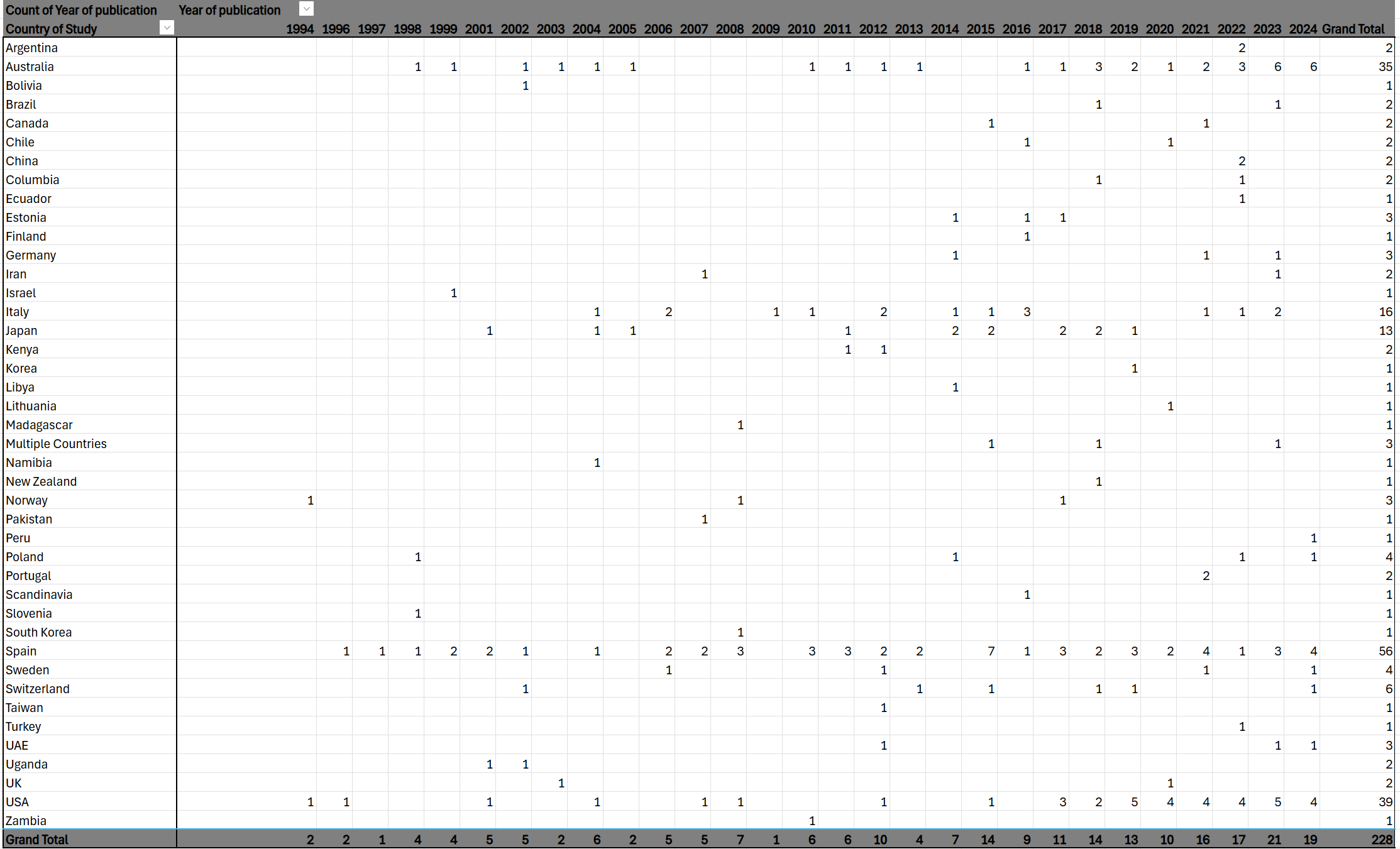


Table S3: The table shows animals from different order which were observed to be affected by sarcoptic mange from 1992-2024


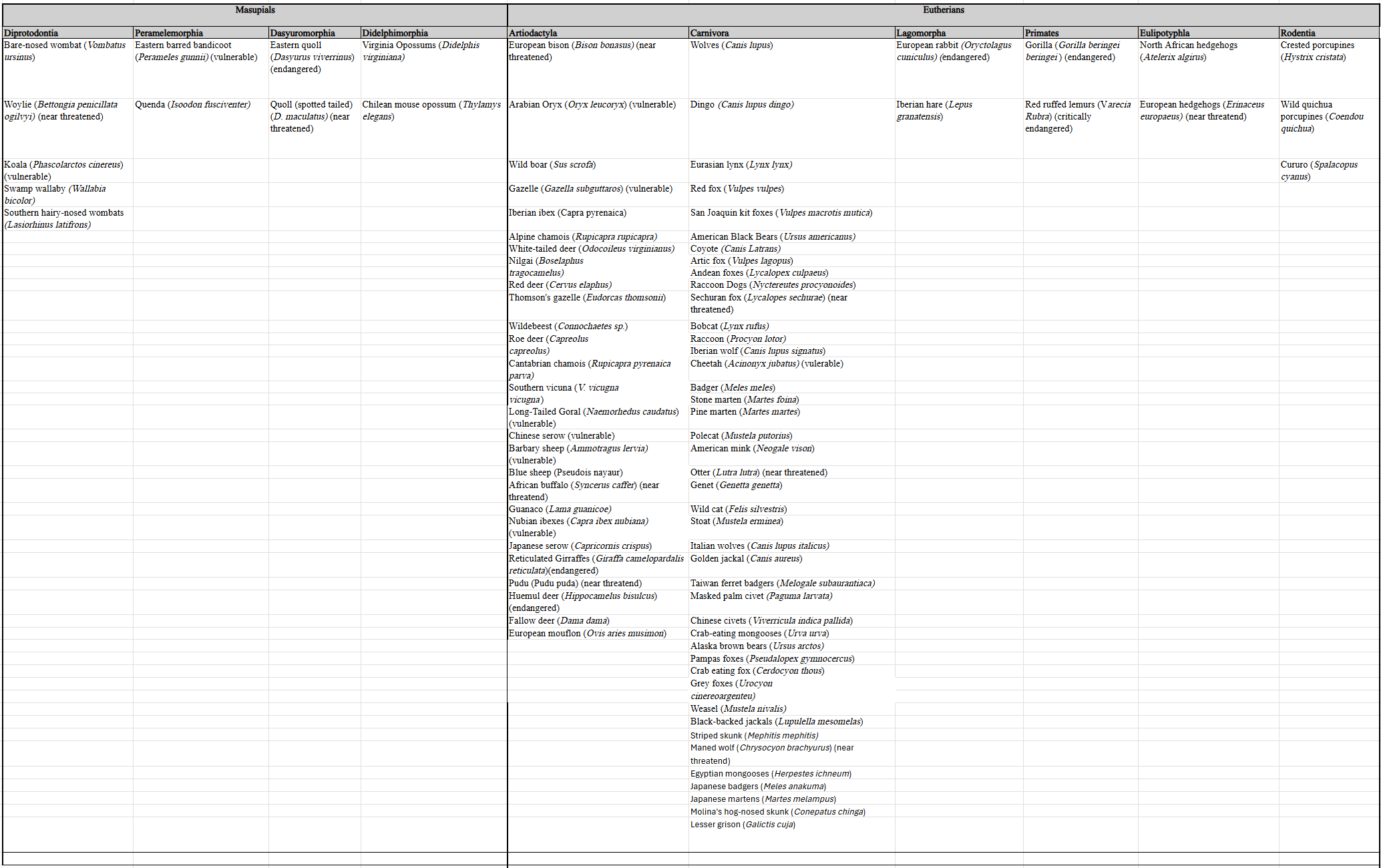


Table S4: The table below lists diagnosis methods applied in 228 studies selected for our analysis.


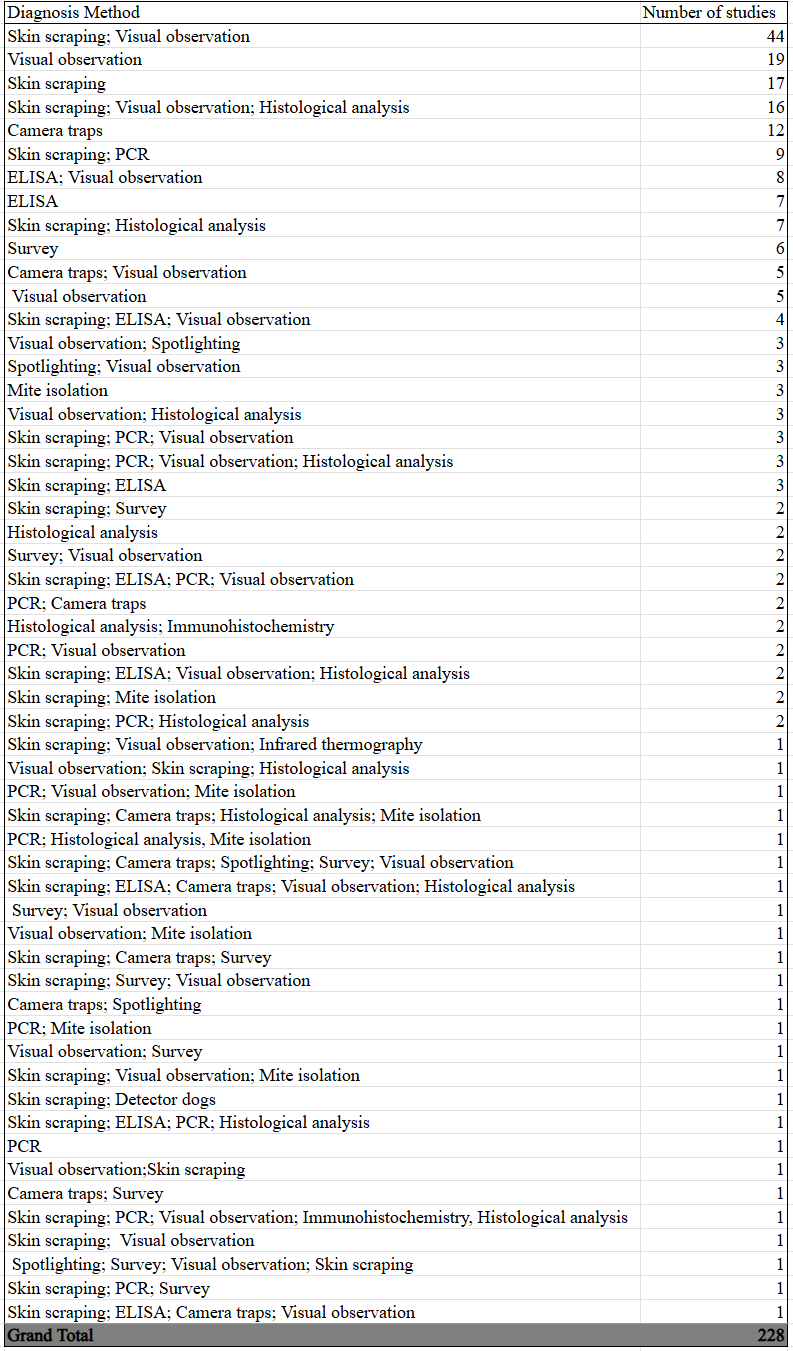


Table S5: Types of study designs:


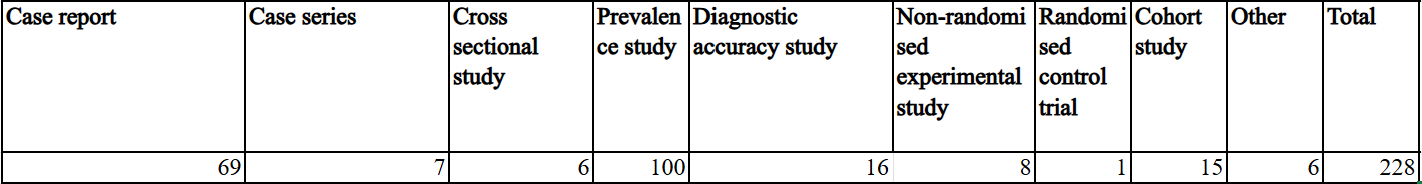


The other category had qualitative research, clinical study, surveillance, comparative experimental and microbiota study.

Table S6: Overview of all studies showing sarcoptic mange detection method:

A: Skin scraping technique

| **Country** | **Population Description** | **Reference** |
| --- | --- | --- |
| Spain | Alpine chamois | Obber et al. 2022 |
| Australia | Bare-nosed wombat | Stannard et al. 2024 |
| Australia | Bare-nosed wombat | Takano, 2024 |
| Spain | Iberian ibex | Valldeperes et al. 2024 |
| USA | American black bears | Tiffin et al. 2024 |
| Peru | Andean fox | Gomez-Puerta et al. 2024 |
| USA | Andean fox, South American gray fox | Millan et al. 2024 |
| Spain | Iberian ibex | Gomez-Guillamon et al. 2024 |
| Australia | Bare-nosed wombat | Wilkinson et al. 2024 a |
| Australia | Bare-nosed wombat | Wilkinson et al. 2024 b |
| UAE | Arabian oryx | Thrivikraman et al. 2023 |
| Germany | Raccoon dog | Klink et al. 2023 |
| Switzerland | Wild boar | Haas et al. 2015 |
| USA | San Joaquin kit fox | Brian et al. 2017 |
| Uganda | Gorilla | Kalema‐Zikusoka et al. 2002 |
| Ecuador | Sechuran fox | Villalba-Briones et al. 2022 |
| Iran | Gazelle | Bazargani et al. 2007 |
| Spain | Spanish ibex | León-Vizcaíno et al. 1999 |
| Spain | Iberian ibex | Serrano et al. 2006 |
| Italy | Alpine chamois | Turchetto et al. 2014 |
| Spain | Iberian ibex | Pérez et al. 2015 |
| Spain | Iberian ibex | Pérez et al. 2016 |
| USA | Feral hog,White-tailed deer, Nilgai, Coyote, Bobcat , Raccoon | Brewster et al. 2017 |
| Spain | Iberian ibex | Espinosa et al. 2017 |
| Spain | Iberian ibex | Castro et al. 2018 |
| Spain | Red deer, Iberian ibex | Iacopelli et al. 2019 |
| USA | American black bear | Niedringhaus et al. 2019 |
| USA | Gray wolf | Brandell, 2021 |
| Spain | Iberian ibex | Pérez et al. 2022 |
| Australia | Bare-nosed wombat | Skerratt et al. 1999 |
| Italy | Chamois | Rambozzi et al. 2004 |
| Australia | Bare-nosed wombat | Hartley & English, 2005 |
| Spain | Iberian ibex | Pérez et al. 2006 |
| Australia | Woylie | Eden et al. 2010 |
| Kenya | Cheetah, Thomson's gazelle, Wildebeest | Gakuya, 2011 |
| USA | Coyote | Wilson et al. 2012 |
| Poland | Red fox, Badger, Wolf, Raccoon dog, Stone marten, Lynx, Pine marten, Polecat, American mink | Kołodziej-Sobocińska, Zalewski & Kowalczyk, 2014 |
| Canada | Coyote | Murray & Edwards, 2015 |
| Estonia | Racoon dog, Red fox | Laurimaa, 2016 |
| Australia | Bare-nosed wombat | Fraser et al. 2018 |
| Spain | Iberian lynx | Oleaga et al. 2019 |
| Italy | Italian wolves | Musto, 2021 |
| Poland | Eurasian lynx | Skorupski 2022 |
| USA | Gray wolf | Verant, Wolf & Romanski 2022 |
| USA | San Joaquin kit fox | Foley et al. 2023 |
| Spain | Cantabrian chamois, Red deer and Roe deer | Fernández-Morán et al. 1997 |
| Spain | Iberian wolves | Domínguez et al. 2008 |
| Japan | Red fox | Uraguchi et al. 2014 |
| Germany | Raccoon | Solís, 2015 |
| Australia | Koala | Speight et al. 2018 |
| Australia | Bare-nosed wombat | Driessen et al. 2021 |
| USA | American black bear | Tiffin, Skvarla & Machtinger 2021 |
| Argentina | Vicugna | Sosa et al. 2022 |
| China | Goral, Takin, Golden monkey, Giant panda, Serows, Wild boars | Wu et al. 2022 |
| Japan | Raccoon Dog | Takahashi et al. 2001 |
| Spain | Spanish ibex | Arenas et al. 2002 |
| Bolivia | Pampas fox, Crab eating fox | Deem et al. 2002 |
| Spain | Barbary sheep | Gonzalez-Candela, León-Vizcaíno & Cubero-Pablo, 2004 |
| Sweden | Red fox | Bornstein et al. 2006 |
| Pakistan | Blue sheep | Dagleish et al. 2007 |
| South Korea | Raccoon Dog | Eo et al. 2008 |
| Zambia | African buffalo | Munang'andu et al. 2010 |
| UAE | Wildebeest | Alasaad et al. 2012 |
| Spain | Wild rabbit | Casais et al. 2015 |
| Switzerland | Badger, Stone marten,  Pine marten, Polecat, Weasel, Stoat, Otter | Akdesir et al. 2018 |
| Korea | Raccoon dogs | Hyun et al. 2019 |
| Germany | Red fox, Stone marten, Racoon dogs | Waindok et al. 2021 |
| Australia | Quenda | Botten, Ash & Jackson, 2022 |
| Argentina | Vicugna, Guanaco | delValleFerreyra et al. 2022 |
| United Arab Emirates | Arabian oryx | Thrivikraman et al. 2023 |
| USA | Red fox | Little, 1996 |
| Israel | Nubian ibex, Gazelle | Yeruham et al. 1999 |
| Poland | Wild boar | Fryderyk et al. 2000 |
| Australia | Bare-nosed wombat | Skerratt et al. 2002 |
| United Kingdom | Red fox | Bates et al. 2003 |
| Australia | Bare-nosed wombat | Skerratt, 2003 |
| USA | Raccoon | Fitzgerald et al. 2004 |
| Namibia | Black-backed jackal | Gowtage-Sequeira et al. 2004 |
| Japan | Raccoon Dog | Noviana et al. 2004 |
| Australia | Bare-Nosed Wombat | Skerratt et al. 2004 |
| Japan | Raccoon dog | Ninomiya & Ogata, 2005 |
| Italy | Fox, Stone marten, Badger | Balestrieri et al. 2006 |
| Spain | Iberian ibex | Alasaad et al. 2008 |
| Fitzgerald 2008 | American black bear | Fitzgerald et al. 2008 |
| Spain | European rabbit | Millan et al. 2010 |
| Spain | European rabbit | Navarro-Gonzalez et al. 2010 |
| Libya | Hedgehog | Hosni & Maghrbi, 2014 |
| Japan | Raccoon dog | Kido et al. 2014 |
| Italy | Roe deer, Badgers, Red fox, Chamois | Angelone-Alasaad et al. 2015 |
| Switzerland, France | Wild boar | Haas et al. 2015 |
| Italy | Red fox | Perrucci et al. 2016 |
| Chile | South American gray fox | Verdugo et al. 2016 |
| Italy | Crested porcupine | Amato et al. 2017 |
| Columbia | Quichua porcupine | Gonzalez-Astudillo et al. 2018 |
| New Zealand | European hedgehog | Kriechbaum et al. 2018 |
| Brazil | Crab-eating fox | Teodoro et al. 2018 |
| USA | American black bear | Niedringhaus et al. 2019 |
| USA | Coyote, San Joaquin kit fox, Red fox | Rudd, 2019 |
| USA | American black bear | VanWick et al. 2019 |
| Spain | Iberian hare | Cardells et al. 2021 |
| Spain | European wildcat | Najera et al.2021 |
| Columbia | Andean porcupine | Busi et al. 2022 |
| Australia | Bare-Nosed Wombat | Naesborg-Nielsen et al. 2022 |
| China | Chinese serow, Goral | Zhou et al. 2022 |
| Iran | Red fox, Rüppell's fox | Akhtardanesh et al. 2023 |
| Spain | Iberian ibex | Fernandez-Munoz et al. 2023 |
| Brazil | Maned wolf | Fiori et al. 2023 |
| Spain | Spanish ibex | Pérez et al. 1999 |
| Uganda | Mountain gorilla | Graczyk et al. 2001 |
| Spain | Spanish ibex | León-Vizcaíno et al. 2001 |
| Spain | Iberian lynx, Red fox, Egyptian mongoose, Common genet, Eurasian badger, Polecat | Millán et al. 2007 |
| Australia | Swamp wallaby | Holz, Orbell & Beveridge 2011 |
| Spain | Iberian ibex | Pérez et al. 2011 |
| Australia | Southern hairy-nosed wombat | Ruykys et al. 2013 |
| Spain | Red fox | Lledó et al. 2015 |
| Japan | Raccoon dog, Raccoon, Japanese badger, Japanese marten, Wild boar, Japanese serow | Makouloutou et al. 2015 |
| Australia | Koala | Speight et al. 2017 |
| Spain | Red fox | Gortázar et al. 1998 |
| Spain | Red deer | Oleaga et al. 2008 |
| Japan | Raccoon dog | Kido et al. 2011 |
| Spain | Iberian wolf | Oleaga et al. 2011 |
| Kenya | Reticulated giraffe | Alasaad et al. 2012 a |
| Italy | Chamois | Alasaad et al 2012 b |
| Spain | Iberian wolf | Oleaga et al. 2015 |
| USA | American Black bear | Peltier et al. 2018 |
| Spain | Iberian ibex | Valldeperes et al. 2019 |
| Chile | Andean fox, Chill fox, Guanaco, Vicuna, Pudu pudu, Yaca, Molin's hog skunk, Huemul deer, Cururo, Lesser grison | Montecino-Latorre et al. 2020 |
| USA | Red fox, Black bear, Coyote | Tiffin et al. 2020 |
| Spain | Iberian ibex, Red deer, Cantabrian chamois, Fallow deer, Roe deer, European mouflon | Moroni 2021 |
| Spain | Wild boar | Valldeperes et al. 2021 |
| Spain | Alpine chamois | Obber et al. 2022 |
| Italy, Switzerland, France | Eurasian lynx, Red fox, Wolf | Moroni et al. 2023 |
| Italy | Wild boar, Red fox | Viani et al. 2023 |

B: Visual observation

| **Country** | **Population Description** | **Reference** |
| --- | --- | --- |
| Australia | Bare-nosed wombat | Casey et al. 2024 |
| Australia | Bare-nosed wombat | Stannard et al. 2024 |
| Australia | Dingo | Meek et al. 2024 |
| Switzerland | Lynx, Red fox | Kuhn et al. 2024 |
| Spain | Iberian ibex | Valldeperes et al. 2024 |
| USA | American black bears | Tiffin et al. 2024 |
| USA | Coyote | Halseth et al. 2024 |
| Sweden | Arctic fox | Wallén et al. 2024 |
| Peru | Andean fox | Gomez-Puerta et al. 2024 |
| USA | Andean fox, South American gray fox | Millan et al. 2024 |
| Spain | Iberian ibex | Gomez-Guillamon et al. 2024 |
| Australia | Bare-nosed wombat | Wilkinson et al. 2024 a |
| Australia | Bare-nosed wombat | Wilkinson et al. 2024 b |
| UAE | Arabian oryx | Thrivikraman et al. 2023 |
| Germany | Raccoon dog | Klink et al. 2023 |
| Italy | Red fox | Franchini et al. 2022 |
| USA | San Joaquin kit fox | Brian et al. 2017 |
| Uganda | Gorilla | Kalema‐Zikusoka et al. 2002 |
| Ecuador | Sechuran fox | Villalba-Briones et al. 2022 |
| USA | Black bear | Houck et al. 2021 |
| Spain | Iberian ibex | Valldeperes et al. 2023 |
| Spain | Spanish ibex | León-Vizcaíno et al. 1999 |
| Itlay | Alpine chamois | Rossi et al. 2006 |
| Spain | Iberian ibex | Serrano et al. 2006 |
| Spain | Iberian ibex | López-Olvera et al. 2015 |
| Spain | Iberian ibex | Pérez et al. 2015 |
| USA | Feral hog,White-tailed deer, Nilgai, Coyote, Bobcat , Raccoon | Brewster et al. 2017 |
| USA | American black bear | Niedringhaus et al. 2019 |
| USA | Gray wolf | Brandell, 2021 |
| Lithuania | Red foxes, Raccoon dogs | Heinicke, 2021 |
| Australia | Bare-nosed wombat | Mayadunnage et al. 2022 |
| Spain | Iberian ibex | Pérez et al. 2022 |
| USA | Coyotes | Grinder & Krausman, 2001 |
| Estonia | Raccoon dog | Suld, 2017 |
| USA | Coyote | Pence et al. 1994 |
| Australia | Bare-nosed wombat | Skerratt et al. 1999 |
| Spain | Iberian ibex | Pérez et al. 2006 |
| Australia | Woylie | Eden et al. 2010 |
| Kenya | Cheetah, Thomson's gazelle, Wildebeest | Gakuya, 2011 |
| USA | Coyote | Wilson et al. 2012 |
| Poland | Red fox, Badger, Wolf, Raccoon dog, Stone marten, Lynx, Pine marten, Polecat, American mink | Kołodziej-Sobocińska, Zalewski & Kowalczyk, 2014 |
| Canada | Coyote | Murray & Edwards, 2015 |
| Estonia | Racoon dog, Red fox | Laurimaa, 2016 |
| Australia | Bare-nosed wombat | Simpson et al. 2016 |
| Spain | Iberian ibex | Espinosa, Granados & Cano-Manuel 2017 |
| Spain | Iberian lynx | Oleaga et al. 2019 |
| Spain | Iberian ibex | Bravo, 2020 |
| Italy | Italian wolves | Musto, 2021 |
| Canada | Coyote | Patter, 2022 |
| Poland | Eurasian lynx | Skorupski 2022 |
| USA | Gray wolf | Verant, Wolf & Romanski 2022 |
| Spain | Gray wolf | Jiménez, Cara & García‐Dominguez, 2023 |
| Spain | Cantabrian chamois, Red deer and Roe deer | Fernández-Morán et al. 1997 |
| Spain | Iberian wolves | Domínguez et al. 2008 |
| Australia | Bare-nosed wombat | Borchard, Eldridge & Wright 2012 |
| Japan | Red fox | Uraguchi et al. 2014 |
| Germany | Raccoon | Solís, 2015 |
| Spain | Iberian ibex | Ráez-Bravo et al. 2016 |
| USA | Gray wolf | Knowles et al. 2017 |
| Australia | Bare-nosed wombat | Driessen et al. 2021 |
| USA | American black bear | Tiffin, Skvarla & Machtinger 2021 |
| Argentina | Vicugna | Sosa et al. 2022 |
| Australia | Bare-Nosed Wombat | Burgess et al. 2023 |
| Spain | Spanish ibex | Arenas et al. 2002 |
| Bolivia | Pampas fox, Crab eating fox | Deem et al. 2002 |
| Switzerland | Lynx | Ryser-Degiorgis et al. 2002 |
| Sweden | Red fox | Bornstein et al. 2006 |
| Pakistan | Blue sheep | Dagleish et al. 2007 |
| Zambia | African buffalo | Munang'andu et al. 2010 |
| Italy | Alpine ibex | RahmanMd et al. 2010 |
| UAE | Wildebeest | Alasaad et al. 2012 |
| Taiwan | Formosan serow | Chen et al. 2012 |
| Switzerland | Badger, Stone marten,  Pine marten, Polecat, Weasel, Stoat, Otter | Akdesir et al. 2018 |
| Korea | Raccoon dogs | Hyun et al. 2019 |
| USA | Coyote | Reddell et al. 2021 |
| Germany | Red fox, Stone marten, Racoon dogs | Waindok et al. 2021 |
| United Arab Emirates | Arabian oryx | Thrivikraman et al. 2023 |
| Norway | Red fox | Overskaug, 1994 |
| USA | Red fox | Little, 1996 |
| Poland | Wild boar | Fryderyk et al. 2000 |
| United Kingdom | Red fox | Bates et al. 2003 |
| USA | Raccoon | Fitzgerald et al. 2004 |
| Namibia | Black-backed jackal | Gowtage-Sequeira et al. 2004 |
| Australia | Bare-Nosed Wombat | Skerratt et al. 2004 |
| Japan | Raccoon dog | Ninomiya & Ogata, 2005 |
| USA | Coyote | Chronert, 2007 |
| Fitzgerald 2008 | American black bear | Fitzgerald et al. 2008 |
| Spain | European rabbit | Millan et al. 2010 |
| Spain | European rabbit | Navarro-Gonzalez et al. 2010 |
| Japan | Raccoon dog | Kido et al. 2014 |
| USA | Gray wolf | Almberg et al. 2015 |
| Scandinavia | Gray wolf | Fuchs et al. 2016 |
| Italy | Red fox | Perrucci et al. 2016 |
| Chile | South American gray fox | Verdugo et al. 2016 |
| Italy | Crested porcupine | Amato et al. 2017 |
| Columbia | Quichua porcupine | Gonzalez-Astudillo et al. 2018 |
| USA | American black bear | Niedringhaus et al. 2019 |
| USA | Coyote, San Joaquin kit fox, Red fox | Rudd, 2019 |
| USA | American black bear | VanWick et al. 2019 |
| Spain | Gray wolf, Red fox, Chamois,  Red deer | Martinez et al. 2020 |
| Spain | European wildcat | Najera et al.2021 |
| Australia | Bare-Nosed Wombat | Old et al. 2021 |
| Australia | Bare-Nosed Wombat | Naesborg-Nielsen et al. 2022 |
| Australia | Bare-Nosed Wombat | Carver et al. 2023 |
| Australia | Bare-Nosed Wombat | Mayadunnage et al. 2023 |
| Uganda | Mountain gorilla | Graczyk et al. 2001 |
| Spain | Spanish ibex | León-Vizcaíno et al. 2001 |
| Spain | Spanish ibex | González-Candela et al. 2006 |
| Spain | Iberian lynx, Red foxes, Egyptian mongooses, Common genets, Eurasian badgers, Polecat | Millán et al. 2007 |
| Madagascar | Red ruffed lemurs | Dutton, Junge & Louis 2008 |
| Australia | Swamp wallaby | Holz, Orbell & Beveridge 2011 |
| Spain | Iberian ibex | Pérez et al. 2011 |
| Sweden | Red fox | Jakubek et al. 2012 |
| Spain | Iberian ibex | Alasaad et al. 2013 |
| Spain | Iberian wolf | Oleaga et al. 2013 |
| Australia | Southern hairy-nosed wombat | Ruykys et al. 2013 |
| Spain | Iberian ibex | Carvalho et al. 2015 |
| Australia | Koala | Speight et al. 2017 |
| USA | American black bear | Rojas-Sereno et al. 2022 |
| Spain | Red fox | Gortázar et al. 1998 |
| Norway | Red fox | Davidson, Bronstein & Handeland 2008 |
| Japan | Raccoon dog | Kido et al. 2011 |
| Spain | Iberian wolf | Oleaga et al. 2011 |
| Kenya | Reticulated giraffe | Alasaad et al. 2012 |
| Switzerland | Red fox | Nimmervoll et al. 2013 |
| Spain | Wild boar | Valldeperes et al. 2021 |
| Spain | Iberian ibex | Valldeperes et al. 2019 |
| Spain | Iberian ibex | Ráez-Bravo et al. 2015 |
| USA | Coyote, Red fox, Gray fox | DeCandia, Leverett & vonHoldt, 2019 |
| USA | San Joaquin kit fox | Riner et al. 2018 |
| Switzerland, France | Wild boar | Haas et al. 2015 |
| Italy | Fox, Stone marten, Badger | Balestrieri et al. 2006 |
| Australia | Bare-nosed wombat | Ringwaldt et al. 2023 |
| USA | Gray wolf | DeCandia et al. 2021 |
| Australia | Bare-nosed wombat | Stannard et al. 2020 |
| Australia | Bare-nosed wombat | Sengupta 2019 |
| Australia | Bare-nosed wombat | Martin et al. 2019 |
| Australia | Bare-nosed wombat | Hartley & English, 2005 |
| Spain | Iberian ibex culled | Sarasa et al. 2010 |
| USA | Red fox | Wails et al. 2024 |
| Spain | Alpine chamois | Obber et al. 2022 |

C: Mange Scoring

| **Country** | **Population Description** | **Reference** |
| --- | --- | --- |
| Australia | Bare-nosed wombat | Stannard et al. 2024 |
| Australia | Dingo | Meek et al. 2024 |
| USA | American black bears | Tiffin et al. 2024 |
| USA | Red fox | Wails et al. 2024 |
| Australia | Bare-nosed wombat | Wilkinson et al. 2024 b |
| Spain | Spanish ibex | León-Vizcaíno et al. 1999 |
| Spain | Iberian ibex culled | Sarasa et al. 2010 |
| Spain | Iberian ibex | López-Olvera et al. 2015 |
| Spain | Iberian ibex | Pérez et al. 2015 |
| USA | Gray wolf | Brandell, 2021 |
| USA | Coyotes | Grinder & Krausman, 2001 |
| USA | Coyote | Pence et al. 1994 |
| Australia | Bare-nosed wombat | Hartley & English, 2005 |
| Canada | Coyote | Murray & Edwards, 2015 |
| Australia | Bare-nosed wombat | Simpson et al. 2016 |
| Spain | Iberian ibex | Espinosa, Granados & Cano-Manuel 2017 |
| Australia | Koala, Bare-nosed wombat, Red fox | Fraser et al. 2018 |
| Australia | Bare-nosed wombat | Martin et al. 2019 |
| Australia | Bare-nosed wombat | Sengupta 2019 |
| Spain | Iberian ibex | Bravo, 2020 |
| Australia | Bare-nosed wombat | Stannard et al. 2020 |
| USA | Gray wolf | DeCandia et al. 2021 |
| Spain | Cantabrian chamois, Red deer, Roe deer | Fernández-Morán et al. 1997 |
| Spain | Iberian wolves | Domínguez et al. 2008 |
| Australia | Bare-nosed wombat | Borchard, Eldridge & Wright 2012 |
| Spain | Iberian ibex | Ráez-Bravo et al. 2016 |
| Zambia | African buffalo | Munang'andu et al. 2010 |
| USA | Coyote | Reddell et al. 2021 |
| Australia | Bare-nosed wombat | Ringwaldt et al. 2023 |
| Italy | Fox, Stone marten, Badger | Balestrieri et al. 2006 |
| USA | Gray wolf | Almberg et al. 2015 |
| Switzerland, France | Wild boar | Haas et al. 2015 |
| Italy | Red fox | Perrucci et al. 2016 |
| USA | San Joaquin kit fox | Riner et al. 2018 |
| USA | Coyote, Red fox, Gray fox | DeCandia, Leverett & vonHoldt, 2019 |
| Australia | Bare-Nosed Wombat | Old et al. 2021 |
| Australia | Bare-Nosed Wombat | Naesborg-Nielsen et al. 2022 |
| USA | Coyote | Reddell et al. 2023 |
| Spain | Iberian ibex | Pérez et al. 2011 |
| Spain | Iberian ibex | Alasaad et al. 2013 |
| Australia | Southern hairy-nosed wombat | Ruykys et al. 2013 |
| Spain | Iberian ibex | Carvalho et al. 2015 |
| Spain | Red fox | Gortázar et al. 1998 |
| Spain | Iberian ibex | Ráez-Bravo et al. 2015 |
| Spain | Iberian ibex | Valldeperes et al. 2019 |
| Switzerland | Red fox | Nimmervoll et al. 2013 |

D: Spotlighting

| **Country** | **Population Description** | **Reference** |
| --- | --- | --- |
| Australia | Bare-nosed wombat | Casey et al. 2024 |
| Australia | Red fox | O'Connor et al. 2021 |
| Australia | Bare-nosed wombat | Sengupta 2019 |
| Australia | Bare-nosed wombat | Stannard et al. 2020 |
| Australia | Bare-nosed wombat | Driessen et al. 2021 |
| Australia | Bare-Nosed Wombat | Burgess et al. 2023 |
| Australia | Bare-nosed wombat | Carver et al. 2023 |
| Australia | Bare-nosed wombat | Mayadunnage et al. 2023 |
| Spain | Red Fox | Gortázar et al. 1998 |

E: Survey

| **Country** | **Population description** | **Reference** |
| --- | --- | --- |
| USA | San Joaquin kit foxes | Cypher et al. 2023 |
| Australia | bare-nosed wombats | Mayadunnage et al. 2024 |
| Italy | Red fox | Franchini et al.2022 |
| Itlay | Alpine chamois | Rossi et al. 2006 |
| Italy | Alpine chamois | Turchetto et al. 2014 |
| Australia | Wombat, Bandicoot (eastern barred), Tasmanian devil, Eagle (wedge tail), Quoll (eastern), Quoll (spotted tailed), Owl (masked), Parrot (swift), Eagle (white bellied sea), Albatross (shy), Parrot (orange-bellied), Pardalote (forty-spotted), Petrel (blue), Albatross (grey headed) | Heathcote et al. 2019 |
| Kenya | Cheetah, Thomson's gazelle, Wildebeest | Gakuya, 2011 |
| Finland | Red fox, Raccoon dog, Badgers | Kauhala, Talvitie & Vuorisalo, 2016 |
| Switzerland | Red fox | Pisano et al. 2019 |
| Canada | Coyote | Patter, 2022 |
| USA | San Joaquin kit foxes | Foley et al. 2023 |
| Australia | Bare-nosed wombat | Driessen et al. 2021 |
| Taiwan | Formosan serow | Chen et al. 2012 |
| Estonia | Red fox | Plumer, Davison & Saarma, 2014 |
| Australia | Bare-nosed wombat | Martin, Handasyde & Skerratt, 1998 |
| Brazil | Spanish ibex | Fiori et al. 2023 |
| Spain | Red fox | Gortázar et al. 1998 |
| Chile | Andean fox, Chill fox, Guanaco, Vicuna, Pudu pudu, Yaca, Molin's hog skunk, Huemul deer, Cururo, Lesser grison | Viani et al. 2023 |

F: Camera traps

| **Country** | **Population Description** | **Reference** |
| --- | --- | --- |
| Poland | European bison, Wolf | Bojarska et al. 2024 |
| USA | Red fox | Wails et al. 2024 |
| Sweden | Artic fox | Wallén et al. 2024 |
| Spain | Red fox | Barroso et al. 2024 |
| Switzerland | Wild boar | Haas et al. 2015 |
| Japan | Raccoon dog | Sugiura et al. 2018 |
| USA | Feral hog, White-tailed deer, Nilgai, Coyote Bobcat, Raccoon | Brewster et al. 2017 |
| Portugal | Iberian wolf, Red fox | Rousseau et al. 2021 |
| Australia | Red fox | O'Connor et al. 2021 |
| Great Britain | Red fox | Scott et al. 2014 |
| Switzerland | Red fox | Pisano et al. 2019 |
| USA | San Joaquin kit fox, Red fox | Cypher et al. 2022 |
| Turkey | Grey wolf, Golden jackal, Red fox | Alper & Anil (2022) |
| USA | San Joaquin Kit fox | Kelly et al. 2022 |
| Spain | Wolf | Jiménez et al. 2023 |
| Australia | Bare-nosed wombat | Borchard 2012 |
| Japan | Raccoon dog | Saito & Sonoda 2017 |
| Australia | Bare-nosed wombat | Driessen et al. 2021 |
| USA | Coyote | Reddell et al. 2021 |
| Australia | Bare-nosed wombat | Ringwaldt et al. 2023 |
| USA | San Joaquin kit fox, Striped skunk, Raccoon, Opossum | Cypher et al. 2023 |
| Brazil | Maned wolf | Fiori et al. 2023 |
| USA | Coyotes | Murray et al. 2021 |
| Spain | Iberian wolf | Oleaga et al. 2011 |
| Italy | Wolf | Galaverni et al. 2012 |
| Norway | Red fox | Sánchez 2017 |

G: PCR

| **Country** | **Population description** | **Reference** |
| --- | --- | --- |
| USA | Red fox | Wails et al. 2024 |
| USA | Andean fox, South American gray fox | Millan et al. 2024 |
| USA | San Joaquin kit fox | Brian et al. 2017 |
| Ecuador | Sechuran fox | Villalba-Briones et al. 2022 |
| Australia | Koala, Bare-nosed wombat, Red fox | Fraser et al. 2018 |
| Argentina | Vicugna | Sosa et al. 2022 |
| China | Goral, Takin, Golden monkey, Giant panda, Serows, Wild boars | Wu et al. 2022 |
| UAE | Wildebeest | Alasaad et al. 2012 |
| Sweden | Wild boar, Sus scrofa | Sanno et al. 2021 |
| Australia | Quenda | Botten, Ash & Jackson, 2022 |
| Spain | Iberian ibex | Alasaad et al. 2008 |
| Italy | Roe deer, Badgers, Red fox, Chamois | Angelone-Alasaad et al. 2015 |
| Chile | South American gray fox | Verdugo et al. 2016 |
| Columbia | Quichua porcupine | Gonzalez-Astudillo et al. 2018 |
| New Zealand | European hedgehog | Kriechbaum et al. 2018 |
| USA | Coyote, Red fox, Gray fox | DeCandia, Leverett & vonHoldt, 2019 |
| Spain | Iberian hare | Cardells et al. 2021 |
| Spain | European wildcat | Najera et al.2021 |
| Columbia | Andean porcupine | Busi et al. 2022 |
| China | Chinese serow, Goral | Zhou et al. 2022 |
| USA | Coyote | Reddell et al. 2023 |
| Spain | Iberian wolf | Oleaga et al. 2013 |
| Italy | Wolf | Galaverni et al. 2012 |
| Japan | Raccoon dog, Raccoon, Japanese marten, Japanese serow, Wild boar | Matsuyama et al. 2019 |
| Chile | Andean fox, Chill fox, Guanaco, Vicuna, Pudu pudu, Yaca, Molin's hog skunk, Huemul deer, Cururo, Lesser grison | Montecino-Latorre et al. 2020 |
| Portugal | Iberian wolf, Wolf, Red fox | Rousseau et al. 2021 |
| Spain | Wild boar | Valldeperes et al. 2021 |
| Italy, Switzerland, France | Eurasian lynx, Red fox, Wolf | Moroni et al. 2023 |
| Italy | Wild boar, Red fox | Viani et al. 2023 |
| USA | American Black bear | Peltier et al. 2018 |

H: ELISA

| **Country** | **Population description** | **Reference** |
| --- | --- | --- |
| Spain | Iberian ibex | Valldeperes et al. 2024 |
| USA | Andean fox, South American gray fox | Millan et al. 2024 |
| Spain | Iberian ibex | Gomez-Guillamon et al. 2024 |
| Spain | European rabbit | Castro-Scholten et al. 2024 |
| Ecuador | Sechuran fox | Villalba-Briones et al. 2022 |
| USA | American Black bear | Houck et al. 2021 |
| Spain | Iberian ibex | Valldeperes et al. 2023 |
| USA | Feral hog, White-tailed deer, Nilgai, Coyote, Bobcat Raccoon | Brewster et al. 2017 |
| Italy | Chamois | Rambozzi et al. 2004 |
| Spain | Iberian lynx | Oleaga et al. 2019 |
| Spain | Cantabrian chamois | Falconi et al. 2010 |
| Spain | Iberian ibex | Ráez-Bravo et al. 2016 |
| USA | Alaska brown bear | Haynes et al. 2023 |
| Sweden | Red fox | Bornstein et al. 2006 |
| Italy | Alpine ibex | RahmanMd et al. 2010 |
| Spain | European rabbit | Millan et al. 2012 |
| Spain | Wild rabbit | Casais et al. 2015 |
| Italy | Wild boar | Villa 2023 |
| Namibia | Black-backed jackal | Gowtage-Sequeira et al. 2004 |
| Switzerland, France | Wild boar | Haas et al. 2015 |
| Scandinavia | Gray wolf | Fuchs et al. 2016 |
| USA | American Black bear | Niedringhaus et al. 2020 |
| Sweden | Red fox | Jakubek et al. 2012 |
| Norway | Red fox | Davidson, Bronstein & Handeland 2008 |
| Spain | Red deer | Oleaga et al. 2008 |
| Spain | Iberian wolf | Oleaga et al. 2011 |
| Spain | Iberian ibex | Ráez-Bravo et al. 2015 |
| Switzerland, France, Italy, Sweden, and Spain | Wild boar | Haas et al. 2018 |
| USA | American Black bear | Peltier et al. 2018 |

I: Histological Analysis

| **Country** | **Population Description** | **Reference** |
| --- | --- | --- |
| Australia | Bare-nosed wombat | Wilkinson et al. 2024 a |
| UAE | Arabian oryx | Thrivikraman et al. 2023 |
| Switzerland | Wild boar | Haas et al. 2015 |
| Uganda | Gorilla | Kalema‐Zikusoka et al. 2002 |
| Iran | Gazelle | Bazargani et al. 2007 |
| Spain | Spanish ibex | León-Vizcaíno et al. 1999 |
| USA | American black bear | Niedringhaus et al. 2019 |
| Spain | Wolf, Red fox, Otter, Pine marten,  Genet,  Wild cat, Stone marten,  Pole cat,  Stoat,  Badger | Oleaga et al. 2018 |
| Spain | Iberian lynx | Oleaga et al. 2019 |
| Germany | Raccoon | Solís, 2015 |
| USA | Gray wolf | Knowles et al. 2017 |
| Australia | Koala | Speight et al. 2018 |
| China | Goral, Takin, Golden monkey, Giant panda, Serows, Wild boars | Wu et al. 2022 |
| Japan | Raccoon Dog | Takahashi et al. 2001 |
| Bolivia | Pampas fox, Crab eating fox | Deem et al. 2002 |
| Pakistan | Blue sheep | Dagleish et al. 2007 |
| South Korea | Raccoon Dog | Eo et al. 2008 |
| Sweden | Wild boar, Sus scrofa | Sanno et al. 2021 |
| Argentina | Vicugna, Guanaco | delValleFerreyra et al. 2022 |
| USA | Red fox | Little, 1996 |
| Slovenia | Chamois | Rode et al. 1998 |
| USA | Raccoon | Fitzgerald et al. 2004 |
| Namibia | Black-backed jackal | Gowtage-Sequeira et al. 2004 |
| Japan | Raccoon Dog | Noviana et al. 2004 |
| Japan | Raccoon dog | Ninomiya & Ogata, 2005 |
| Fitzgerald 2008 | American black bear | Fitzgerald et al. 2008 |
| Spain | Red deer, Chamois, Wolf, Red fox, Roe deer | Oleaga et al. 2012 |
| Italy | Red fox | Perrucci et al. 2016 |
| Italy | Alpine Chamois | Salvadori et al. 2016 |
| Chile | South American gray fox | Verdugo et al. 2016 |
| Italy | Crested porcupine | Amato et al. 2017 |
| Columbia | Quichua porcupine | Gonzalez-Astudillo et al. 2018 |
| Brazil | Crab-eating fox | Teodoro et al. 2018 |
| Spain | Gray wolf, Red fox, Chamois,  Red deer | Martinez et al. 2020 |
| Spain | European wildcat | Najera et al.2021 |
| Columbia | Andean porcupine | Busi et al. 2022 |
| Uganda | Mountain gorilla | Graczyk et al. 2001 |
| Australia | Swamp wallaby | Holz, Orbell & Beveridge 2011 |
| Australia | Koala | Speight et al. 2017 |
| Spain | Iberian wolf | Oleaga et al. 2011 |
| Switzerland | Red fox | Nimmervoll et al. 2013 |
| USA | American Black bear | Peltier et al. 2018 |
| Spain | Wild boar | Valldeperes et al. 2021 |

J: Radio telemetry

| **Country** | **Population description** | **Reference** |
| --- | --- | --- |
| Australia | Dingo | Meek et al. 2024 |
| USA | San Joaquin kit foxes | Cypher et al. 2023 |
| Estonia | Raccoon dog | Suld, 2017 |
| USA | Coyote | Wilson et al. 2012 |
| Canada | Coyote | Murray & Edwards, 2015 |
| Canada | Coyote | Patter, 2022 |
| Switzerland | Eurasian lynx | Schmidt-Posthaus et al. 2002 |
| Norway | Red fox | Overskaug, 1994 |
| USA | Gray wolf | Almberg et al. 2015 |
| USA | Coyote | Reddell et al. 2023 |

K: Mite isolation

| **Country** | **Population Description** | **Type** | **Reference** |
| --- | --- | --- | --- |
| Switzerland | Wild boar | Mite isolation | Haas et al. 2015 |
| Poland | European bison, Wolf | Sedimentation and direct floatation method | Bojarska et al. 2024 |
| Switzerland | Lynx | Mite isolation | Ryser-Degiorgis et al. 2002 |
| Sweden | Wild boar, Sus scrofa | Post frozen method | Sanno et al. 2021 |
| Australia | Bare-nosed wombat | Mite isolation | Skerratt et al. 2002 |
| Australia | Bare-Nosed Wombat | Mite isolation | Skerratt et al. 2004 |
| Italy | Free ranging animals | Syringe Eppendorf method | Soglia 2009 |
| Spain | Iberian wolf | Post frozen method | Oleaga et al. 2013 |
| Japan | racoon dogs | Post frozen method | Matsuyama 2015 |
| Japan | Raccoon dog, Raccoon, Japanese marten, Japanese serow, Wild boar | Post frozen method | Matsuyama et al. 2019 |
| Spain | Iberian ibex, Red deer, Cantabrian chamois, Fallow deer, Roe deer, European mouflon | Post frozen method | Moroni 2021 |
| Japan | Racoon dog | Microtube method | Kido 2017 |

L: Historical Data Analysis

| **Country** | **Population Description** | **Reference** |
| --- | --- | --- |
| Switzerland | Red fox | Pisano et al. 2019 |
| Germany, Austria | Chamois | Fuchs, Deutz & Gressmann, 2000 |
| USA | Red Fox, Gray foxes | Kelly & Sleeman, 2003 |
| Scandinavia | Gray wolf | Fuchs et al. 2016 |
| USA | American black bear | Rojas-Sereno et al. 2022 |
| Sweden | Red fox | Willebrand et al. 2022 |
| Switzerland | Badger, Stone marten,  Pine marten, Polecat, Weasel, Stoat, Otter | Akdesir et al. 2018 |

M: Immunohistochemistry

| **Country** | **Population Description** | **Reference** |
| --- | --- | --- |
| Spain | Red deer, Chamois, Wolf, Red fox, Roe deer | Oleaga et al. 2012 |
| Italy | Alpine Chamois | Salvadori et al. 2016 |
| Spain | Wild boar | Valldeperes et al. 2021 |
