# Supplementary material for: A comprehensive systematic review of sarcoptic mange diagnostic methods in wildlife
Source: PeerJ. 2026 Jul 29;14:e21609. doi: 10.7717/peerj.21609 (PMC13428544; doi:10.7717/peerj.21609)
Supplement: Supplemental Information 3 [file peerj-14-21609-s003.docx]

Intended audience

Our systematic review on sarcoptic mange detection methods is aimed at researchers and wildlife health authorities. It emphasizes that animal behavior and environmental conditions are critical in selecting appropriate detection methods, especially in remote areas. The analysis also outlines the strengths and limitations of each method, guiding both research design and effective management strategies.
